# Y chromosome variation and prostate cancer ancestral disparities

**DOI:** 10.1016/j.isci.2025.112437

**Published:** 2025-04-15

**Authors:** Pamela X.Y. Soh, Alice Adams, M.S. Riana Bornman, Jue Jiang, Phillip D. Stricker, Shingai B.A. Mutambirwa, Weerachai Jaratlerdsiri, Vanessa M. Hayes

**Affiliations:** 1 Ancestry and Health Genomics Laboratory, Charles Perkins Centre, School of Medical Sciences, Faculty of Medicine and Health, University of Sydney, Camperdown, NSW 2006, Australia; 2 Faculty of Science, University of Bath, BA2 7AY Bath, UK; 3 School of Health Systems and Public Health, University of Pretoria, Pretoria, South Africa; 4 St Vincent’s Prostate Cancer Research Centre, Sydney, NSW 2010, Australia; 5 Department of Urology, Sefako Makgatho Health Science University, Dr George Mukhari Academic Hospital, Medunsa, Ga-Rankuwa, South Africa; 6 Manchester Cancer Research Centre, University of Manchester, M20 4GJ Manchester, UK

**Keywords:** Human genetics, Sequence analysis, Cancer

## Abstract

Prostate cancer (PCa) is marked by significant ancestral bias, with African men disproportionately impacted. However, genome profiling studies have yet to explore the mutational landscape and disparity contribution of the male-determining Y chromosome. Using a cohort of 106 African and 57 European PCa cases, biased toward aggressive presenting primary disease, we performed complete Y chromosome interrogation for inherited and somatic variance. Capturing unexplored early-diverged Y-haplogroup substructure, while European men are 3.1-fold more likely to present with a rare potentially deleterious germline variant, a higher proportion of African patients acquired Y chromosome tumorigenic events (26.4% African, 14% European). While somatic copy number alterations were universally more common to aggressive tumors, besides shared alterations impacting *DDX3Y* and *USP9Y*, African derived tumors were prone to somatic losses associated with *KDM5D*, *PCDH11Y*, and *RBMY*. This much-needed African inclusive study alludes to possible Y chromosome contribution, at least in part, to treatment resistance and worsened mortality rates in African men.

## Highlights

• Phylogenetic analysis showed Y-haplogroup refinement is needed among African lineages
• Different potentially deleterious germline SNVs in *TBL1Y*/*UTY* in both populations
• Somatic CN loss in tumor suppressor *DDX3Y* in both African and European tumors
• Treatment resistance-associated *KDM5D* somatic CN loss increased in African tumors

## Graphical abstract

**Figure:**
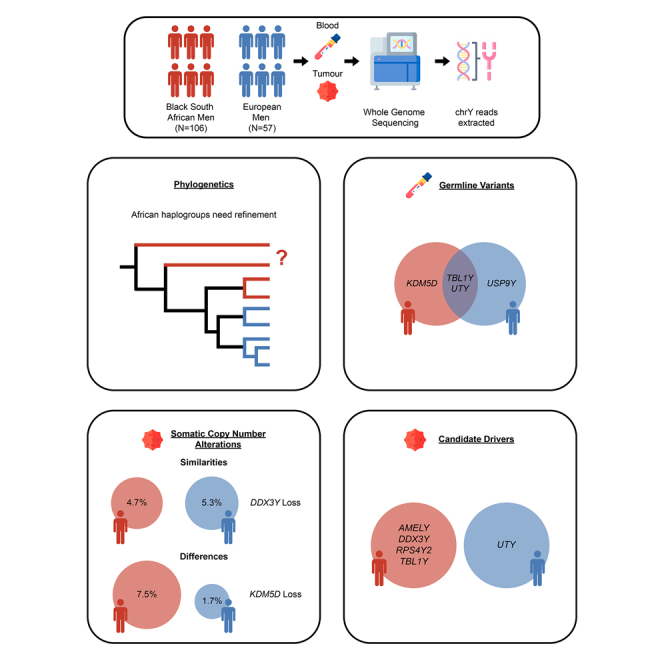

## Introduction

The human Y chromosome (chrY) is small and gene-deficient, while rich in repeat elements and segmental duplications, with chromosomal-wide copy number variation (CNV).^1^ As a consequence of extensive structural complexity, chrY is notoriously difficult to sequence,^2^^,^^3^ and largely ignored in genome-wide disease interrogation studies.^4^ Except for the pseudoautosomal region (PAR),^5^ the lack of recombination leads to Y-haplotypes, including haplogroup-specific Y-CNVs,^6^^,^^7^ which are passed on from father to son as patrilineages with high geographic and ethnic specificity.^8^ In a similar fashion, prostate cancer (PCa) is highly heritable,^9^ such that a diagnosed father and brother with PCa doubles familial associated risk,^10^ while ancestry is a significant risk factor for PCa presentation, age at onset, and associated mortality.^11^ The commonality of inheritance and ethnic specificity provides the rationale for further interrogation of the “underappreciated” male chromosome.

Typically excluded from genome-wide PCa association studies,^12^ targeted chrY explorations have provided a glimpse into geo-ethnic specific associations. While data from men of European, Ashkenazi Jewish,^13^ African American,^14^ and Korean^15^ ancestry showed no significant association between Y-haplotypes and PCa, conversely, studies have identified increased PCa risks for Y-haplogroup DE (DE-M145; Japanese),^16^ O3 (O-M122; Japanese),^14^ I1c (I-Z17943; Swedish),^17^ and microvariant alleles of short tandem repeats in DYS388 (Malaysian), DYS439 (Malaysian),^18^ and DYS458 (Portuguese).^19^ Additionally, extensive genealogical data from Utah in the United States has identified 7.3% of 1000 unique Y chromosomes to be associated with high risk of PCa.^20^ Although presenting with the highest global mortality rates,^21^ data from Africa is lacking. In prostate tumor cell lines, chrY gene loss is common in high grade LNCaP and PC3,^22^^,^^23^ while loss of the entire chrY, a frequent event among cancer types, is rare in PCa but is associated with poor progression-free survival.^24^^,^^25^ However, the specific contributors of inherited and acquired chrY variation between ethnicities remains unclear.

From our previously generated whole genome data focused on autosomal interpretation,^26^ here, we compared blood and tumor chrY data between 106 African and 57 European men with histopathologically confirmed treatment naive primary PCa. Biased toward aggressive disease, patient inclusion required a confirmed genetic ancestry as non-admixed, with data generation and analysis reliant on a single technical and informatic pipeline. As such, we minimized for inter-study sequencing associated artifacts, while captured through our focus on southern African men, not only the highest global PCa mortality rates,^21^ but also the greatest genetic diversity.^27^

## Results

### ChrY phylogenetics and uncaptured African ancestral sublineages

**Figure 1:**
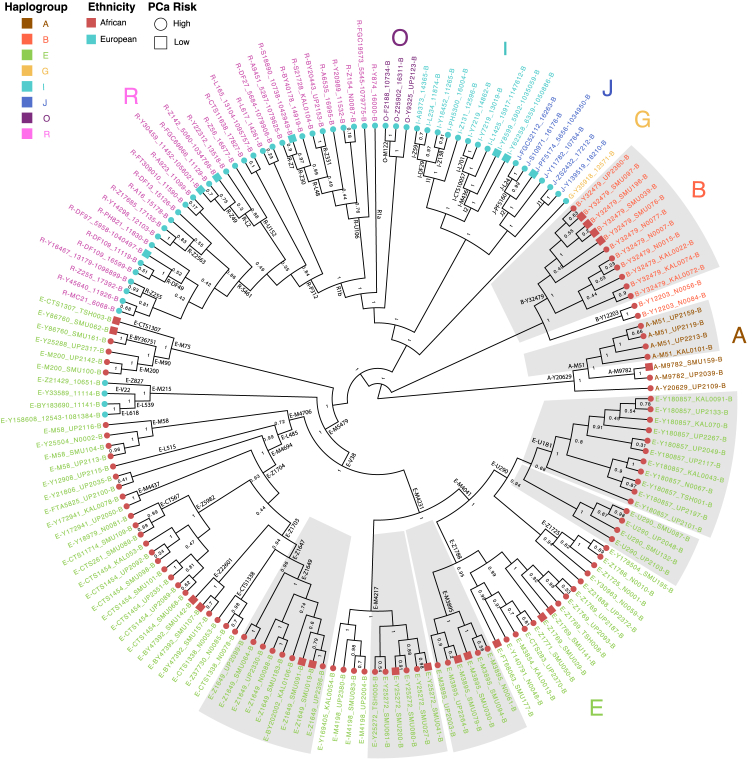
Phylogenetic tree of Y chromosome sequences (midpoint-rooted) Ethnicities are indicated by the tip colors (light blue = European, red = African), with the tip shape indicating high or low risk prostate cancer (square = low risk, circle = high risk), and sample names are colored according to predicted haplogroups. Values on the branches indicate bootstrap values. Lineages shaded in gray boxes indicate regions of the tree where samples with the same predicted haplogroups may have uncaptured sublineages as there is high bootstrap support for their split.

Phylogenetic analysis of our patient cohort showed broad Y-haplogroup representation including the African predominant E (81.1%, 86/106) and specific A and B, and European-specific R (58%, 33/57), G, I, J and O (Figure 1; Table S1). It was not surprising that our southern African subjects presented with the earliest diverged Y-haplogroups A and B, representing the root-region for contemporary modern humans’ paternal evolutionary tree.^7^ Furthermore, notable within-chrY phylogenetic sub-branching suggests significant uncaptured Y-haplogroup diversity across southern Africa. This includes four (of seven) currently unknown A-M51, or International Society of Genetic Genealogy (ISOGG) Y-DNA database (https://isogg.org/tree/, version 15.73, last revised 11 July 2020) A1b1b2a sequences, which splits into two additional nodes (bootstrap value = 1, 4 individuals) from A-M9782 (bootstrap value = 1). Eleven (of 13) currently unknown B-Y32479 sequences, a subgroup of B-M150 (descending from ISOGG B2a1a1a1a2), split into two major groups (bootstrap value = 1, 5 and 6 individuals each) with further sub-branching. Haplogroup E showed high bootstrap support for uncaptured African-specific subbranches within E-Z1649 (9 individuals), E-M4217 (6), E-M3895 (6), E-U290 (4) and E-U181 (11).

**Figure 2:**
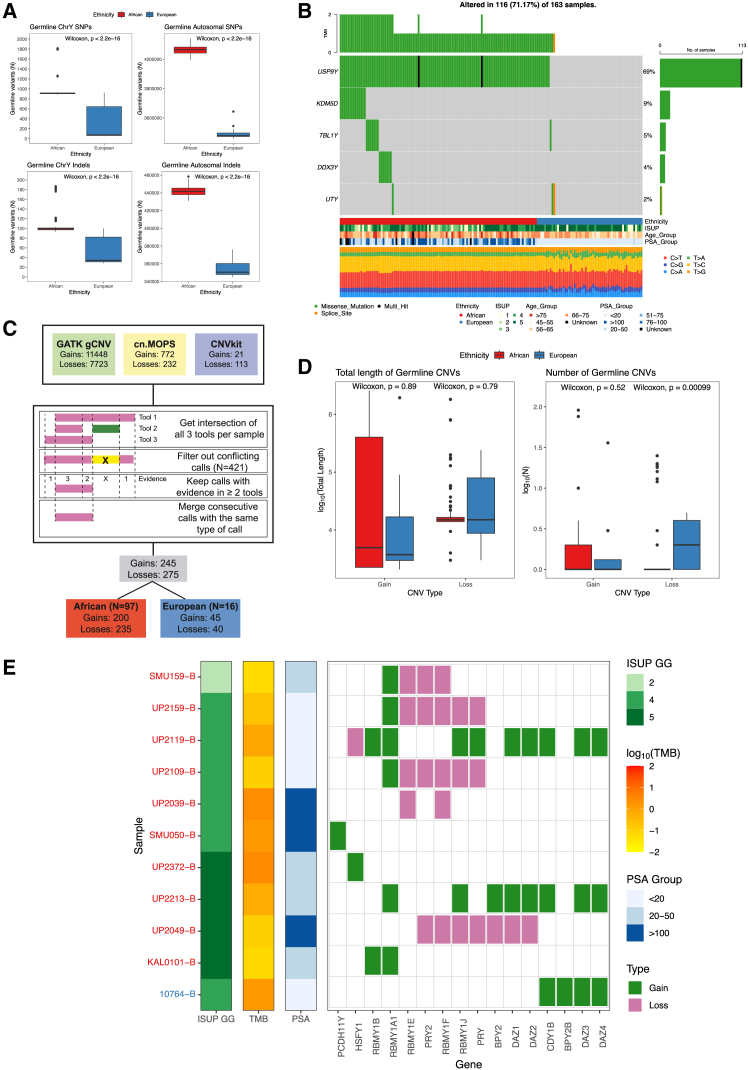
A higher number of germline single nucleotide variants (SNVs) were found in men of African ancestry compared to European ancestry, while among protein-coding genes, germline copy number variation (CNV) was limited in men of European ancestry (A) Boxplots of the number of germline single nucleotide variants (SNVs) and indels in chrY compared to the autosome^26^ by ethnicity. Data are represented by the median (horizontal line in box), the interquartile range (IQR) from 25^th^ to 75^th^ percentiles (box), with whiskers extending to the largest or smallest values no more than 1.5 times the IQR. (B) Oncoplot displaying the number of non-synonymous germline variants found in the population. (C) Workflow for the germline CNV analysis, showing the number of calls found by each tool and the method used to merge calls. (D) The total length and the number of germline CNVs with evidence in at least two tools between ethnicities. Data are represented by the median (horizontal line in box), the IQR from 25^th^ to 75^th^ percentiles (box), with whiskers extending to the largest or smallest values no more than 1.5 times the IQR. (E) CNV losses and gains identified in protein coding genes. Sample names are on the y-axis, color coded by ethnicity (red, African; blue, European). Clinical features for each sample are indicated by the first three columns, including the International Society of Urological Pathology grade group (ISUP GG), tumor mutational burden in the autosome^26^ (TMB; values are in number of mutations per megabase), and prostate specific antigen group (PSA; values are in ng/mL).

Overall, African over European men had a significantly greater number of chrY single nucleotide variants (SNVs, mean 1010 vs. 332) and small insertions and deletions (indels, 106 vs. 53.8), which mirrors the autosomal means (4296320 vs. 3432990 and 442130 vs. 353398, respectively) (Figure 2A; Wilcoxon rank-sum test, *p* < 2.22e-16 for all). However, unlike autosomal whole genome data where Africans had greater within-population variance (SNVs interquartile range (IQR): 61234 vs. 36715), the opposite is true for chrY variance (SNVs IQR: 14.8 vs. 580) (Figure 2A). The retracted chrY variance observed is consistent with our predominantly Bantu cohort (75.47%, 80/106 from E-V38 lineages), that would have descended from a limited pool of male migrants (and thus limited Y-lineages) into Southern Africa during the Bantu migration into the region approximately 1500 years ago.^28^ While the A-M51 sublineages appear to be restricted to indigenous southern African San peoples, and B-M150 is a mix between Bantu and San peoples,^29^^,^^30^ it is notable that all individuals self-identified ethno-linguistically as southern Bantu. Through autosomal interrogation we further confirm contributing San population fractions for all haplogroup A (mean 0.248, range 0.096–0.495) and haplogroup B (mean 0.153, range 0.0166–0.249) individuals, while 24.4% (21/86) of haplogroup E representative individuals lacked a San ancestral fraction (<0.1%) (Figure S1).

Four European patients shared the E-ancestral sub-branch E-M215 (and sublineage E-M35), with three patients with the E-M78 lineage (sublineage of E-L539) which predominate in Northern Africa, Eastern Africa, the Near East and Europe^31^ (Figure 1; Table S1). In addition, these four patients shared a nonsynonymous *USP9Y* variant rs7067496 which was present in all African patients (Figure 2B; Table S2). This variant is the M235 marker for haplogroup F in ISOGG. All seven African patients in haplogroup A shared a nonsynonymous *DDX3Y* variant rs111406208 (not in ISOGG database), while all 13 African patients in haplogroup B shared a nonsynonymous *KDM5D* variant rs35681523 (marker M152 for haplogroup B-Y10267).

### No associations between Y-haplogroups and PCa risk

Notable distinctions between the multi-ethnic cohorts include average age at diagnosis for African men being 5.2 years later (66.9 vs. 61.7 years old), while the European cohort was biased toward slightly more advanced disease at surgery defined here as International Society of Urological Pathology group grading (ISUP) 3, 4 or 5 or high-risk PCa (HRPCa, 87.7%, 50/57 vs. 83%, 88/106). As previously presented for the Southern African Prostate Cancer Study (SAPCS)^32^^,^^33^ and in contrast to the National Cancer Consortium Network (NCCN) European-driven criteria that includes within the HRPCa definition as having a Prostate Specific Antigen (PSA) level >20 ng/mL, our African patients present with mean PSA levels 12.8-fold greater (255.94 ng/mL, range 4.28–4847 ng/mL). Unable to use European-derived PSA criteria to classify HRPCa in our study, we restrict our analyses to ISUP grading.

Testing for the association between major haplogroups of patients with HRPCa (*N* = 138) versus low-risk PCa (LRPCa; *N* = 25), we found no significant association between major haplogroups and aggressive PCa risk (*p* = 0.8, Fisher’s exact test) (Table S3). Splitting into ethnicities, there was no significant association between haplogroups and PCa risk in European (LRPCa *N* = 7, HRPCa *N* = 50, *p* > 0.9, Fisher’s exact test), nor African patients (LRPCa *N* = 18, HRPCa *N* = 88, *p* = 0.3, Fisher’s exact test). Using a ‘treeWAS’ approach,^34^ we further tested for the association between 9448 biallelic genotypes and HRPCa versus LRPCa while correcting for the population structure within the phylogenetic tree, and found no significant associations even at a relaxed *p*-value threshold of 0.1.

### Potentially deleterious germline chrY variants

A total of 13 nonsynonymous germline SNVs were detected through ANNOVAR, including 6 in *USP9Y*, 2 each in *KDM5D*, *TBL1Y* and *UTY*, and 1 in *DDX3Y* (Figure 2B; Table S2). None of these variants had an available ClinVar prediction. Notably, the three variants common (allele frequencies (AF) > 0.66) to our Southern Africans (rs373532788 *TBL1Y*, rs111406208 *DDX3Y*, rs35681523 *KDM5D*), are rare in the largely west African ancestral gnomAD populations (AF < 0.012), while the single variant common to our European cohort (rs7067496 *USP9Y*; AF = 0.07) is fixed in Southern Africans (AF = 1). Filtering for variants that were damaging/possibly damaging in at least one of SIFT, SIFT4G, PolyPhen2 HDIV or PolyPhen2 HVAR, which predicts the functional impact of variants through evolutionary conservation and changes to protein structure and are not dependent on other prediction tools,^35^^,^^36^ followed by filtering out variants predicted to be benign by InterVar, eight potentially deleterious variants (PDVs) were left. Variants were considered unknown (38.5%, 5/13; 3 African and 2 European) if not found in the gnomAD v4.1 database, which has genomic variants from over 800,000 individuals across the globe.^37^

Five European patients presented with a rare PDV each and included *TBL1Y* (known chrY:7074584 A>G; present in both African/African American and European samples in gnomAD at AF < 6.1e-05), three *USP9Y* (unknown chrY:12842454 C>T, unknown chrY:12859400 A>C, and known rs766658730 C>G) and *UTY* (known rs200431840 G>A). These five European patients had ISUP grades of 4 or 5, with PSA levels ranging from 8.6 to 17 ng/mL, and were diagnosed between 58 and 72 years old. Two unknown African-specific rare PDVs identified include *UTY* (chrY:13355115 T>C) and *KDM5D* (chrY:19744477 T>A). The African patient with the *UTY* PDV was diagnosed at 62 years old, 4.9 years earlier than the mean African age of diagnosis, with a PSA of 1232.8 ng/mL and ISUP grade of 5. The African patient with the *KDM5D* PDV was diagnosed at 75 years, with a PSA of 51 ng/mL and ISUP grade of 4. Notably, the common Southern African *TBL1Y* (rs373532788, AF = 0.066) variant showed deleterious predictions from PolyPhen2 HDIV, PolyPhen2 HVAR, MutationTaster, fathmm-MKL coding, and with high CADD (22.2) and DANN (0.997) scores. While this study is not designed for common variant interrogation, the potential for this candidate PDV to contribute to PCa risk in this population warrants further investigation.

### Germline chrY CNVs are common to African patients

We utilized three tools for germline CNV analysis, namely GATK gCNV,^38^ cn.MOPS^39^ and CNVkit^40^ (Figure 2C). Requiring two-caller concurrence, we identified 200 gains and 235 losses in 97 (91.5%) African and 45 gains and 50 losses in 16 (28%) European patients (Figure S2). While there were no significant differences between the total length of CNV gains or losses between ethnicities, men of African ancestry had significantly fewer number of losses (Figure 2D, *p* = 0.00099, Wilcoxon rank-sum test).

CNVs impacted 15 and 4 protein-coding and 42 and 14 RNA genes in African and European samples, respectively (Figures S3 and S4). Notably, 83% (88/106) of African men presented with CN-loss spanning the lncRNA *TTTY22,* which included all patients presenting with haplogroups A (*n* = 7) and E-V38 (*n* = 80), and a single E-M75 patient, while 74.5% (79/106) showed loss of lncRNA *ENSG00000228379*, including 6/7 haplogroup A, 72/80 E-V38 and one E-M75 (Figure S3). While a single European patient showed CN-loss in both RNA genes, another presented with *TTTY22* CN-gain. Excluding these likely ancestral events, CNVs were present in 40 genes in 13.2% (14/106) of African and 12 genes in 14% (8/57) of European patients.

Notably, no losses were detected among protein-coding genes in Europeans, however, a single HRPCa patient showed CN-gain in 4 genes: *BPY2B*, *CDY1B*, *DAZ3* and *DAZ4* (Figures 2E and S4). Among African patients, CNVs in protein-coding genes were present in only one LRPCa patient (1/27; 3.7%), and nine HRPCa patients (9/79; 11.4%). All seven chrY’s from haplogroup A had at least one germline CNV, with six carrying a gain in *RBMY1A1*, and four loss in *RBMY1E* and *RBMY1F*. Three haplogroup E African derived chrY’s showed germline CNVs including gain of *HSFY11*, gain of *PCDH11Y*, and CN-loss spanning *PRY2*, *RBMY1F*, *RBMY1J*, *PRY*, *BPY2*, *DAZ1*, and *DAZ2*. The most frequent losses in African patients were in *RBMY1F* (5 patients), *PRY2* (4 patients) and *RBMY1E* (4 patients), while the most common gain was in *RBMY1A1* (6 patients).

### Somatic copy number alterations disproportionately impact African tumors

**Figure 3:**
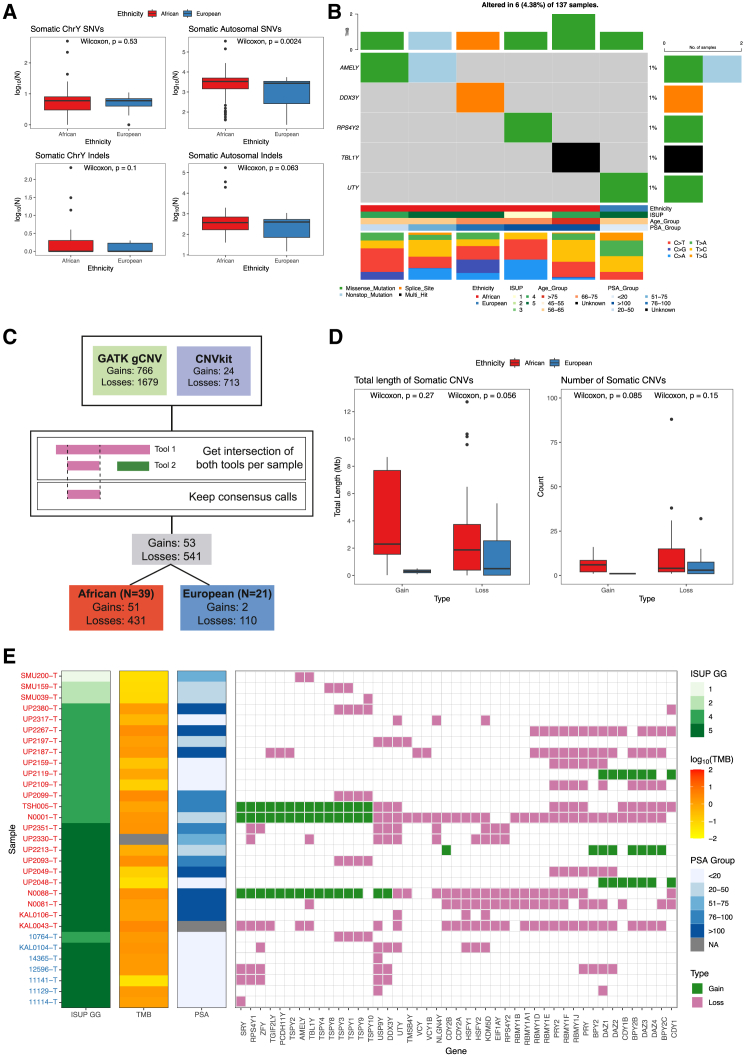
Somatic nonsynonymous variants were exceedingly rare and copy number gains were absent in European tumors, however copy number losses were common to both African (19.8%) and European (12.3%) tumors (A) Boxplots of the number of somatic single nucleotide polymorphisms (SNVs) and indels in chrY compared to the autosome^26^ by ethnicity. Data are represented by the median (horizontal line in box), the interquartile range (IQR) from 25^th^ to 75^th^ percentiles (box), with whiskers extending to the largest or smallest values no more than 1.5 times the IQR. (B) Oncoplot displaying the number of non-synonymous somatic variants found in the population. (C) Workflow for the somatic CNV analysis, showing the number of calls found by each tool and the method used to merge calls. (D) The total length and number of somatic CNVs found with evidence in both tools between ethnicities. Data are represented by the median (horizontal line in box), the IQR from 25^th^ to 75^th^ percentiles (box), with whiskers extending to the largest or smallest values no more than 1.5 times the IQR. (E) Somatic CNV losses and gains identified in protein coding genes. Sample names are on the y-axis, color coded by ethnicity (red, African; blue, European). Clinical features for each sample are indicated by the first three columns, including the International Society of Urological Pathology grade group (ISUP GG), tumor mutational burden in the autosome^26^ (TMB; values are the log_10_ number of mutations per megabase), and prostate specific antigen group (PSA; values are in ng/mL).

Unlike germline small variants, the number of chrY somatic small variants were not significantly different between ethnicities, contrasting against the autosome, where there was a significantly greater number of somatic SNVs in Africans than Europeans (*p* = 0.0024) (Figure 3A). Nonsynonymous mutations were found in the genes *AMELY*, *DDX3Y*, *RPS4Y2*, *TBL1Y* in African tumors (5 samples: 4 HRPCa 1 LRPCa), and in *UTY* in one European HRPCa tumor (Figure 3B; Table S4). The tumor sample from the African patient UP2330, which had extremely high tumor mutational burden (TMB) of 174.85 mutations/Mb in the autosome, had a nonstop variant in *AMELY* as well as a splice site variant in *DDX3Y*.

Using the intersection and consensus between GATK gCNV^38^ and CNVkit^40^ for somatic copy number alterations (SCNAs), we identified 51 and 2 gains and 431 and 110 losses in 39 African and 21 European derived tumors, respectively, and representing 36.8% of each cohort (Figures 3C and S5). There were no significant differences (*p* > 0.05) between the total length and number of SCNAs between ethnicities (Figure 3D). SCNAs were present in 45 and 17 protein-coding and 121 and 41 RNA genes in African and European tumors, respectively (Figures S6 and S7). The most frequent losses in African tumors impacted the RNA genes *ENSG00000236951*, *ENSG00000291034*, *RBMY2FP*, *RNU6-1314P* and *TTTY5* (10/106 samples each; 9.4%), while the most common loss in European tumors was also in *ENSG00000291034* (6/57 samples; 10.5%). CN-gain was only observed in three RNA genes in European tumors (one sample each), compared to gains in 78 RNA genes in African tumors (mean 2.46 samples per gene, range 1–3) (Figure S6).

Among protein-coding genes, while no gains were detected in European tumors, there was an elevated frequency of SCNAs for African tumors, though not significant (22.6% vs. 12.3%, *p*-value = 0.143, Fisher’s test). *USP9Y* loss was the most common SCNA (5 patients, 8.7%). For African tumors, gains were detected in 3 samples for 18 genes each, whereas the most common loss impacted *PRY*, *PRY2*, and *RBMY1F* (10 patients for all, 9.4%) (Figures 3E and S7). LRPCa tumors had few SCNAs, representing 11% (3/27) of African tumors only, with losses in 1–3 protein-coding genes. For HRPCa tumors, SCNAs were more frequent in African (21/106; 19.8%) over European tumors (7/57, 12.3%), although not significant (*p* = 0.2792, Fisher’s exact test). However, African tumors displayed SCNAs in a higher number of protein-coding genes (mean = 12.9, SD = 11.3, range 3–42), compared to European tumors (mean = 4.14, SD = 3.08, range 1–9), more than 3-fold higher despite the nearly 2-fold difference in sample size.

### Lack of significant loss of chrY (LOY)

As an estimate for Y ploidy, the mean depth in X-degenerate regions of chrY were divided by half the mean depth of whole-genome coverage as previously reported.^26^ We identified a median Y ploidy of 0.842 and 0.841 in the blood of African and European men, respectively, with minimum values of 0.535 and 0.558 in these populations (Figure S8). Y ploidy was not significantly associated with age in African men (R^2^ = 0.01, *p*-value = 0.237), while age was a significant effect in European men but also similarly had high variability (R^2^ = 0.07, *p*-value = 0.04) (Figure S9). Conversely, median somatic Y ploidy was 0.831 and 0.847, with minimum 0.479 and 0.639 in African and European men, respectively (Figure S10). While there was no significant association with age for both ancestries (Figure S11), when calculating the difference between tumor and blood Y ploidy per sample we observed no significant LOY (Figure S12). However, a single African patient (UP2351) presenting at age 66 year with ISUP grade 5 diagnosed PCa and PSA 92 ng/mL showed a greater than 4 times SD from the mean of ploidy loss in the tumor (0.36), suggesting at least partial somatic LOY.

## Discussion

In this study, we sought to explore the landscape of germline and somatic Y chromosome variance associated with PCa ancestral disparities within an African-inclusive cohort.

### Y-haplogroups

While some Y-haplogroups have been associated with increased PCa risk,^16^^,^^17^^,^^18^^,^^19^^,^^20^ others have not.^13^^,^^14^^,^^15^ Limited by our case-only design and sample size for risk association analyses, we found no correlation with high-risk disease. However, the identification of unknown African-specific chrY sub-branches will enable future studies focused on inclusion across the African diaspora, including modern human’s earliest diverged paternal lineages (haplogroups A and B). Additionally, we provide evidence for the possible incorrect assignment of chrY F-M235 as haplogroup defining, as it was present in all African patients (haplogroups A, B, and E) and four European E-M35 patients.

The haplogroup-specific variation at *TTTY22* lncRNA, commonly experiencing CN-loss in E-P177 (E1b), is rare in all other haplogroups.^41^ Here we found *TTTY22* loss in the majority of our African samples, including all seven haplogroup A Y chromosomes from the A1b lineage (A-M51, A-M9782, A-Y20629) absent from the previous study,^41^ and in a single sample presenting with the likely non-Bantu E-M75 (E2)^30^ lineage. We also refine *TTTY22* loss in E-P177 as a marker for sub-lineage E-V38 (Bantu African) but not E-M215 (European). Overall, these results show that *TTTY22* loss is likely ancestral to modern humans, with gain of this lncRNA a more recent event in human evolutionary divergence.

### Germline variants

Seeking to identify inherited chrY PCa predisposing variants in this “gene-poor chromosome”, of the 13 nonsynonymous variants, only the likely benign *USP9Y* rs7067496 was shared between the ancestries. Its commonality to all African participants again suggests this variant to be ancestral. Notably, European men were 3.1-fold more likely than African men to present with a rare PDV (prevalence 8.7% vs. 2.8%) and more likely to present with a PDV impacting *USP9Y* or *TBL1Y*, rather than *UTY* or *KDM5D*. This difference can be attributed to the “Out-of-Africa” bottleneck, where populations that have migrated out of Africa accumulate a higher number of deleterious mutations due to less time for purifying selection compared to African populations.^42^ Of the eight PDVs identified, four are unknown (including two African) and three are found in *USP9Y* (prevalence of 5.3% of Europeans). While *USP9Y* overexpression has been shown to inhibit lung cancer tumorigenesis,^43^ USP9Y fusion to TTTY15 appears to be a common event in prostate tumors of Chinese ancestral patients,^44^ suggesting a possible mechanism for PDV functionality through altering USP9Y expression or TTTY15 fusion. Conversely, *TBL1Y* has not been reported to play a role in cancers but rather cardiac development and hearing loss, however, gene expression is primarily in the prostate and cochlea in adults.^45^^,^^46^ *KDM5D*, a histone H3 lysine 4 demethylase, can modify gene expression resulting in aggressive PCa, interacts with the androgen receptor (AR) and can alter sensitivity to docetaxel.^47^^,^^48^ *UTY* is another lysine demethylase that plays a role a prostate differentiation by mediating the interaction between NKX3.1 and G9a, with the authors suggesting a disruption to this network could potentially result in PCa predisposition.^49^ Further work will be required to investigate whether these African PDVs alter gene function or epigenetic regulation.

While CNV is known to vary between populations,^50^ individual specific CNV events were infrequent in our study. A single European HRPCa patient presenting with germline CN-gains impacting *CDY1B*, *DAZ3*, and *DAZ4*, also showed patient-specific gain in *BPY2B*. Notably, loss of *BPY2B* has been associated with male infertility.^51^ In African patients, CNVs included a gain in *RBMY1A1* (5.7% of patients), and losses in *RBMY1F*, *RBMY1E*, *RBMY1J*, *PRY*, and *PRY2* (2.8–4.7%). *RBMY1* genes are known to be prone to CNV and structural organization, particularly CN-gains,^52^ so it is notable that loss predominates for three of these family protein coding genes. While both the *RBMY1* and *PRY* genes have been suggestively linked to infertility,^53^^,^^54^ and *RBMY1* has no known effects in cancer, intriguingly, *PRY* has been linked to apoptosis of spermatids and spermatozoa.^53^

### Somatic variants

Here, we found nonsynonymous variants impacting *AMELY*, *DDX3Y*, *RPS4Y2*, and *TBL1Y* in five African tumors (4.7%), and *UTY* in a single European tumor (1.8%), highlighting the potential for these chrY genes as ancestry-specific cancer driver candidates. The higher frequency in the African tumors provides potential rationale for the inclusion of chrY in health disparity studies.

In contrast to somatic SNVs, prostate tumor SCNAs have been reported in a single study identifying a high frequency (14–52%) of gene losses impacting *SRY*, *ZFY*, *BPY1*, *KDM5D*, *RBM1*, and *BPY2*.^22^ Additionally, for PCa cell lines losses in *NLGN4Y*, *TMSB4Y*, *TSPY1*, and *TTTY13* have been observed for LNCaP and PC3, with further *DDX3Y*, *TTTY15*, *USP9Y*, and *UTY* losses in PC3 only.^23^ Studies have also noted differential expression of the chrY genes in PCa cell lines and regulation can be affected by androgen.^55^^,^^56^ Here, SCNAs were notably absent from European LRPCa and rare in African low-risk tumors (up to three genes in three patients). Observing no distinct pattern of SCNAs among HRPCa, while gains were limited to African tumors, the most frequent protein-coding events were losses in *PRY*, *PRY2*, *RBMY1F*, *RBMY1J*, *UTY*, *DAZ1*, and *KDM5D* (>7.5% of African patients), with notable lack of CNs for *PRY2*, *RBMY1F*, and *RBMY1J* in European tumors. Overall, SCNAs were present in almost three times as many genes in African tumors (166 RNA and protein-coding genes) than European tumors (58 genes), partially owing to the larger African cohort, but may suggest differing transcriptional profiles between the ancestries.

Additionally, we found *DDX3Y* and *USP9Y* CN-loss to impact both African (5 and 6 tumors, respectively) and European tumors (3 and 5, respectively). However, one African tumor also showed a CN-gain of *DDX3Y* and *USP9Y*. *DDX3Y* along with its paralog on the X chromosome, *DDX3X*, are involved in RNA regulation with roles in neurogenesis, and can function as tumor suppressors or oncogenes that have been implicated in other cancers.^57^ Recent work has also found that DDX3Y is stabilized by USP9Y, and tumor suppressive effects were observed in lung cancer cells when both DDX3Y and USP9Y were overexpressed.^43^

Conversely, among many of the differences between both ancestries, notable differences were in *KDM5D*, *PCDH11Y* and *RBMY* genes. As highlighted above, *KDM5D* has been associated with aggressive PCa and resistance to docetaxel.^47^^,^^48^ Here, we identified eight HRPCa African tumors and one HRPCa European tumor to carry a somatic loss in *KDM5D*. Furthermore, three African tumors had CN-gain in *PCDH11Y*, while one African tumor had CN-loss, and alterations in this gene were absent among European tumors. Expression of PCDH11Y has been noted to induce Wnt signaling and promote androgen-resistant malignant growth in the LNCaP cell line.^58^ Lastly, *RBMY* genes losses were common in African tumors but absent from the European tumors. *RBMY* genes have shown a role in hepatocellular carcinoma and is potentially involved in AR activity regulation.^59^^,^^60^ Collectively, the differences in SCNA between these genes in African versus European tumors point toward pathways for treatment-resistant tumors in African patients, suggesting that these genes may contribute to worsened mortality rates in African men.^21^

While LOY, and/or partial LOY, appears to be a frequent event in cancers,^24^ yet rare in PCa, conversely, its presence has been associated with poor PCa survival.^24^^,^^25^ Observing no significant ancestry-derived differences in inherited or somatic LOY, a single African outlier showed partial LOY in his aggressive presenting tumor. Besides seminal work by Qi et al. having explored LOY across many cancers using largely European-derived resources (>80% European in The Cancer Genome Atlas (TCGA) cohort),^24^ future and larger African inclusive studies are required to determine if ancestral differences in complete or partial LOY contribute, at least in part, to the observed clinical disparities.

### Conclusions

In conclusion, identifying as yet unknown chrY haplogroup substructure representing modern humans’ earliest diverged paternal lineages, while we found no association between haplogroups and clinical features, European patients were 3.1-fold more likely to present with a rare PDV with ethnically driven gene specificity. In turn, we identified both commonalities (*DDX3Y* and *USP9Y*) and differences (*KDM5D*, *PCDH11Y*, and *RBMY* genes) in acquired chrY variation between African and European patients. Conversely, while European tumors lacked CN-gains, prevalence for SCNA in protein-coding genes was elevated for African tumors (22.6% vs. 12.3%). Identifying here disparities in the patients’ inherited profile and acquisition of tumor-derived variance in men of African and European ancestries calls for further interrogation and inclusion of the understudied chrY in genomic studies aimed at developing an ancestrally inclusive model for PCa precision medicine.

### Limitations of the study

While African inclusive, yet conscious to provide a technically, analytically, and as close to possible clinicopathologically matched non-African data source, there are several limitations that need to be highlighted. Compared with non-African focused efforts, this study is small making it hard to provide definitive conclusions. While biased toward aggressive disease, the late presentation of African patients makes it difficult to prioritize early-onset disease for the identification of PDVs, while lack of PCa awareness in the study region^61^ would further limit prioritization for family history, a known PCa risk factor. Furthermore, lack of publicly available known African-relevant pathogenic chrY variants current databases, as well as lack of population and regionally matched genomic data, further limits our ability to adequately predict functionality and association. Additionally, due to the nature of merging CNVs across results from different tools, we were also unable to report specific numbers of gains and losses due to variation between callers, however, we attempted to capture the presence of a CNV that was in concordance among the callers.

## Resource availability

### Lead contact

Further information and requests for resources should be directed to and will be fulfilled by the lead contact, Professor Vanessa M. Hayes (vanessa.hayes@sydney.edu.au).

### Materials availability

This study did not generate new unique reagents.

### Data and code availability

• Data: DNA sequence data have been deposited at the European Genome-Phenome Archive (EGA), and the accession number is listed in the key resources table. They are available upon request if access is granted. This paper uses existing, publicly available data from gnomAD v3.1.2. The link to the dataset is listed in the key resources table.
• Code: This paper does not report original code.
• All other items: Any additional information required to reanalyze the data reported in this paper is available from the lead contact upon request.

## Acknowledgments

We acknowledge, most importantly, the patients and the clinical staff who contributed to the Southern African Prostate Cancer Study (SAPCS) and the St Vincent’s Hospital Sydney resources in South Africa and Australia, respectively, as well as all the additional authors who contributed to generating or interrogating the published whole genome data resource. We acknowledge the Sydney Informatic Hub at the University of Sydney for providing critical computational infrastructure. Genomic sequencing was supported by the 10.13039/501100000925National Health and Medical Research Council (NHMRC) of Australia through a Project Grant (APP1165762 to V.M.H.) and Ideas Grants (APP2001098 to V.M.H. and M.S.R.B.; APP2010551 to V.M.H.). Further analytics was supported by a U.S.A. Congressionally Directed Medical Research Programs (CDMRP) Prostate Cancer Research Program (PCRP) HEROIC Consortium Award (PC210168 and PC230673, HEROIC PCaPH Africa1K to V.M.H. and M.S.R.B., which includes co-leads Professors Gail Prins, University of Illinois at Chicago, U.S.A., and Mungai Peter Ngugi, University of Nairobi, Kenya), a U.S.A. National Institute of Health (NIH) 10.13039/100000054National Cancer Institute (NCI) Award (1R01CA285772-01 to V.M.H.) and a U.S.A. Prostate Cancer Foundation (PCF) Challenge Award (2023CHAL4150 to V.M.H.). V.M.H. was further supported by the 10.13039/501100002337Petre Foundation via the University of Sydney Foundation, Australia.

## Author contributions

P.X.Y.S. and V.M.H. conceived and designed the study. P.X.Y.S., A.A., J.J., and V.M.H. provided methodological support and/or performed analyses, while P.X.Y.S. led the formal investigations. M.S.R.B., P.D.S., S.B.A.M., W.J., and V.M.H. contributed key resources. P.X.Y.S., A.A., and V.M.H., wrote and edited the manuscript, P.X.Y.S. generated the figures, while V.M.H. provided supervision, project administration, and funding. All authors reviewed and approved the final manuscript.

## Declaration of interests

V.M.H. is a Member of Active Surveillance Movember Committee and received an honorarium from The Korean Urological Oncology Society for 2024 Annual Conference as a guest speaker.

## STAR★Methods

### Key resources table

**Table:** 

REAGENT or RESOURCE	SOURCE	IDENTIFIER
**Deposited data**		

DNA sequence data	Jaratlerdsiri et al.^26^	EGA: EGAS00001006425
gnomAD v3.1.2 HGDP + 1KGP subset	Karczewski et al.^62^ (gnomAD)	https://gnomad.broadinstitute.org/downloads#v3-hgdp-1kg
San reference population DNA sequence data	W.J., unpublished data	N/A
GRCh38 strict callability mask, version 20160622	1000 Genomes Project; Poznik et al.^63^	https://ftp.1000genomes.ebi.ac.uk/vol1/ftp/data_collections/1000_genomes_project/working/20160622_genome_mask_GRCh38/
Reference hg38 chrY fasta	UCSC	https://hgdownload.cse.ucsc.edu/goldenpath/hg38/chromosomes/chrY.fa.gz
Canonical annotations GENCODE V46	UCSC	https://genome.ucsc.edu/cgi-bin/hgTables

**Software and algorithms**		

bcftools v1.17	Danecek et al.^64^	https://www.htslib.org/download/
samtools v1.6	Danecek et al.^64^	https://www.htslib.org/download/
Y-leaf v3.0	Ralf et al.^65^	https://github.com/genid/Yleaf
GATK v4.2.0.0 HaplotypeCaller	Poplin et al.^66^	https://github.com/broadinstitute/gatk
vcf-tab-to-fasta perl script	Jinfeng Chen	https://github.com/JinfengChen/vcf-tab-to-fasta
Muscle5 v5.1	Edgar^67^	https://github.com/rcedgar/muscle
RAxML-ng v1.0.3	Kozlov et al.^68^	https://github.com/amkozlov/raxml-ng
FigTree v1.4.4	Rambaut	https://github.com/rambaut/figtree/
Adobe Illustrator 2023	Adobe Inc.	https://www.adobe.com/au/products/illustrator.html
R package ‘treeWAS’	Collins and Didelot^34^	https://github.com/caitiecollins/treeWAS/
R package ‘vcfR’	Knaus and Grunwald^69^	https://github.com/knausb/vcfR
R package ‘ape’	Paradis and Schliep^70^	https://cran.r-project.org/web/packages/ape/index.html
PLINK v2.0	Chang et al.^71^	https://www.cog-genomics.org/plink/2.0/
ADMIXTURE v1.3.0	Alexander et al.^72^	https://dalexander.github.io/admixture/
pong v1.5	Behr et al.^73^	https://dalexander.github.io/admixture/
ANNOVAR	Wang and Hakonarson^74^	https://annovar.openbioinformatics.org
InterVar (online database, version 13 June 2022)	Li and Wang^75^	https://wintervar.wglab.org
GATK v4.4.0.0 gCNV	Poplin et al.^66^	https://github.com/broadinstitute/gatk
R package ‘cn.MOPS’	Klambauer et al.^39^	https://bioconductor.org/packages/cn.mops/
CNVkit v0.9.10	Talevich et al.^40^	https://github.com/etal/cnvkit
R package ‘bedtoolsr’	Patwardhan et al.^76^	https://github.com/PhanstielLab/bedtoolsr
R package ‘IRanges’	Lawrence et al.^77^	https://bioconductor.org/packages/IRanges
R package ‘ggplot2’	Wickham^78^	https://ggplot2.tidyverse.org

### Experimental model and study participant details

The primary publication from Jaratlerdsiri et al.^26^ contains all patient information and genomic data used for this study, including a total 163 male patients of European (*n* = 57) and African ancestry (*n* = 106).

### Method details

Unless otherwise stated, RStudio v4.3.1 was used to generate plots (using ‘ggplot2’ package^78^).

#### Patient cohort and genomic data

DNA was extracted from blood and prostate tumour tissue samples from 163 male patients with histopathologically diagnosed PCa (mean age 65.2 ± 8.12) of European (*n* = 57, including 53 from Australia and 4 from South Africa) and African ancestry (*n* = 106, from South Africa). Processing of 150 bp paired end Illumina NovaSeq data into analysis-ready bam files (aligned to the hg38 reference genome), as well as ancestry classification, was conducted previously.^26^ Aggressive disease was defined using the International Society of Urological Pathology Grade Group (ISUP GG) into high- (ISUP GG ≥ 3; HRPCa) and low-risk (ISUP GG < 3; LRPCa) PCa. Prostate serum antigen (PSA) levels were described previously.^26^

#### Y-chromosome haplogroup and phylogenetic analyses

Y chromosome reads were extracted from germline analysis-ready bam files using samtools v1.6.^64^ Y-haplogroups were predicted using Y-leaf v3.0 using bam files as input.^65^ Germline variants were called using GATK’s v4.2.0.0 program HaplotypeCaller^66^ in haploid mode to produce vcf files. As a large portion of the Y chromosome is inaccessible, the GRCh38 strict callability mask (20160622 version) from 1000 Genomes Project (1KGP) was used to filter variants (https://ftp.1000genomes.ebi.ac.uk/vol1/ftp/data_collections/1000_genomes_project/working/20160622_genome_mask_GRCh38/).^63^

Using SNVs present in these Y-chromosome callable regions, the vcf file was converted to fasta format using perl scripts from https://github.com/JinfengChen/vcf-tab-to-fasta (accessed 20 Sep 2023), then aligned using Muscle5 v5.1.^67^ RAxML-ng v1.0.3 was then used to create a maximum likelihood phylogenetic tree, using the GTRGAMMA model with 200 bootstraps with the Felsenstein bootstrap and transfer bootstrap expectation options.^68^ FigTree v1.4.4 (http://tree.bio.ed.ac.uk/software/figtree/) was used for visualisation using midpoint rooting. Adobe Illustrator 2023 was used to further colour and annotate the figure.

The R package ‘treeWAS’^34^ was used to test for associations between PCa risk and biallelic variants while accounting for population structure in a phylogenetic tree. The vcf file stated above was filtered to keep 9448 biallelic variants, then the R package ‘vcfR’^69^ was used to read the file into RStudio and to extract genotypes. The genotypes were then converted to a matrix (base R function). The best tree file from RAxML-ng’s output was read into R using the ‘ape’ package.^70^ The treeWAS function was run with default settings with the best tree, the genotype matrix, and a vector of phenotypes for each sample (1 = HRPCa, 0 = LRPCa). As the default p-value threshold is 0.01, the function was also tested using relaxed p-value thresholds of 0.05 and 0.1.

#### Autosomal substructure

A set of 77,372 genome-wide exomic variants that we previously successfully used to discern ancestral differences within-Africa^79^ were extracted from the autosomal data of our cohort^26^ using bcftools v1.17,^64^ along with reference populations of Asian (Han Chinese, CHB), European (Utah, USA residents with North and Western European ancestry, CEU), East African (Luhya Kenyan, LWK) and West African (Yoruba Nigerian, YRI) ancestry (N=20 each, randomly selected) from the gnomAD v3.1.2 HGDP and 1KGP subset.^62^ An additional 20 individuals with known San ancestry were added to the reference population (W.J., unpublished data). The data was merged with bcftools v1.17^64^ and converted to PLINK^71^ format, subsequently SNVs that were not in dbSNP156 and those that were fixed were removed, leaving 64,654 autosomal SNVs. Unsupervised ADMIXTURE v1.3.0 analysis was performed on this dataset for K=2 to K=10 with five-fold cross-validation (CV), with ten replicates each,^72^ and runs were evaluated for concordance using pong v1.5.^73^ While K=3 provided the lowest CV error rate at a mean 0.253, here, the average fractions at K=4 was reported instead as it displays the split between South Africa and East/West Africa ancestries, with only slightly higher mean CV error rate of 0.255.

#### Annotations and SNV predicted effects

ANNOVAR was used to annotate variants for databases RefSeq gene from UCSC (refGene; 20211019 version), dbSNP (avsnp150; 20170929 version), REVEL (revel; 20161205 version), ClinVar (clinvar_2022123; 20230105 version), and dbNSFP (dbnsfp42a; 20210710 version).^74^ Nonsynonymous variants were queried on InterVar (last update 13 June 2022) to determine pathogenicity.^75^

#### Copy number variation (CNV) analysis

As other studies have shown,^80^^,^^81^ the best approach to calling CNVs in whole genome sequencing data is to combine several callers. However, many of these callers do not adjust for the haploid nature of chrY and even fewer are able to detect somatic copy number alterations (SCNAs) in chrY. As such, we selected tools that can handle chrY data, including two tools GATK v4.4.0.0 gCNV^38^ and cn.MOPS v1.46.0^39^ that were suggested to be among the top four callers by Gabrielaite and colleagues,^81^ as well as CNVkit v0.9.10.^40^

GATK’s gCNV was run with default parameters according to online tutorials provided by GATK.^38^ For the germline analysis, read counts were first collected according to a default bin length of 1000 for each sample (PreprocessIntervals, CollectReadCounts), including a hg38 chrY fasta file downloaded from UCSC (https://hgdownload.cse.ucsc.edu/goldenpath/hg38/chromosomes/chrY.fa.gz). Then, the chrY callability mask was used to annotate and filter intervals (AnnotateIntervals, FilterIntervals). Contig ploidy was then determined (DetermineGermlineContigPloidy), followed by CNV calling in cohort mode (GermlineCNVCaller). Finally, copy number segments and sample results were consolidated with PostprocessGermlineCNVCalls.

For the somatic analysis, read counts were collected from the tumour bam files, then a panel of normal (PoN) was created from the germline read counts (CreateReadCountPanelOfNormals). Tumour read counts were then standardised and denoised against the PoN (DenoiseReadCounts). Next, the germline vcf of SNVs were converted to an interval list, and reference and alternate allele counts were individually collected for tumour and blood bam files (CollectAllelicCounts). Contiguous copy ratios were then grouped into copy number segments (ModelSegments), specifying tumour and normal allelic counts. Finally, copy number netural, amplified or deleted segments were called with CallCopyRatioSegments and plotted with PlotModeledSegments.

For cn.MOPS, the callability mask was read into R v4.2.2 and converted into a GRanges object. All chrY blood bam files were then read into R using the function “getSegmentReadCountsFromBam”, while specifying the callability mask GRanges object. Germline CNV calling was then conducted by using the “haplocn.mops” function with default parameters, which conducts poisson normalisation across all samples and does “DNAcopy” circular binary segmentation. Copy numbers were then calculated using the “calcIntegerCopyNumbers” function, then returning the CNVs and CNV regions (CNVR) using the “cnvs” and “cnvr” functions respectively. As cn.MOPS does not currently have a haploid pipeline for somatic CNVs, this was not conducted.

For CNVkit, germline CNV calling was conducted using the batch pipeline on blood chrY bam files, specifying the whole genome method (-m wgs), Hidden-Markov Model germline segmentation (--segment-method hmm-germline), a target average size of 1000 (to match GATK gCNV’s default bin length), specifying the callability mask as an access file, the hg38 chrY fasta from UCSC, and known canonical annotations (GENCODE V46) downloaded from UCSC’s Table Browser (including primary tables kgXref and knownAttrs). For somatic CNV calling, the same batch pipeline was used with the same parameters except tumour chrY bam files were used as input, with blood chrY bam files specified under “--normal", and the tumour version of the Hidden-Markov Model segmentation was used (--segment-method hmm-tumour).

For each sample, we used the multi-intersect function from the ‘bedtoolsr’ package^76^ to get the intersection between calls across the tools. Conflicting calls were then filtered out, and calls were kept if there was evidence of a CNV in at least two tools for the germline analysis, while for the somatic analysis, calls found in both CNVkit and GATK gCNV were kept. Lastly, consecutive calls with the same type of call (gain or loss) were merged using the reduce function from the ‘IRanges’ package.^77^

#### Loss of chrY (LOY) analysis

Using X-degenerate regions as defined previously,^3^ the ‘depth’ function from samtools v1.6 was used to calculate depth of coverage at each base position. For each sample, an estimated Y chromosome ploidy was calculated by dividing the mean coverage across these X-degenerate regions by half the mean autosomal coverage as reported in our previous publication.^26^

### Quantification and statistical analysis

Wilcoxon rank sum and Fisher’s exact tests were conducted in R v4.3.1. Statistical details of these tests are provided in the figure or table legends, or in the text. A p-value < 0.05 was considered statistically significant.

## Supplemental information

Document S1. Figures S1–S12 and Table S2–S4

Table S1. Y haplogroups (Hg) of the cohort as determined by Yleaf
