# Supplementary material for: Y chromosome variation and prostate cancer ancestral disparities
Source: iScience. 2025 Apr 15;28(5):112437. doi: 10.1016/j.isci.2025.112437 (PMC12124675; doi:10.1016/j.isci.2025.112437)
Supplement: Document S1. Figures S1–S12 and Table S2–S4 [file mmc1.pdf]

iScience, Volume *28*

Supplemental information

Y chromosome variation and prostate cancer ancestral disparities

Pamela X.Y. Soh, Alice Adams, M.S. Riana Bornman, Jue Jiang, Phillip D. Stricker, Shingai B.A. Mutambirwa, Weerachai Jaratlerdsiri, and Vanessa M. Hayes

Y-chromosomal variation and prostate cancer racial disparities

Pamela X.Y. Soh, Alice Adams, M.S. Riana Bornman, Jue Jiang, Phillip D. Stricker, Shingai B.A. Mutambirwa, Weerachai Jaratlerdsiri, Vanessa M. Hayes

SUPPLEMENTARY DOCUMENT

Supplementary Figures

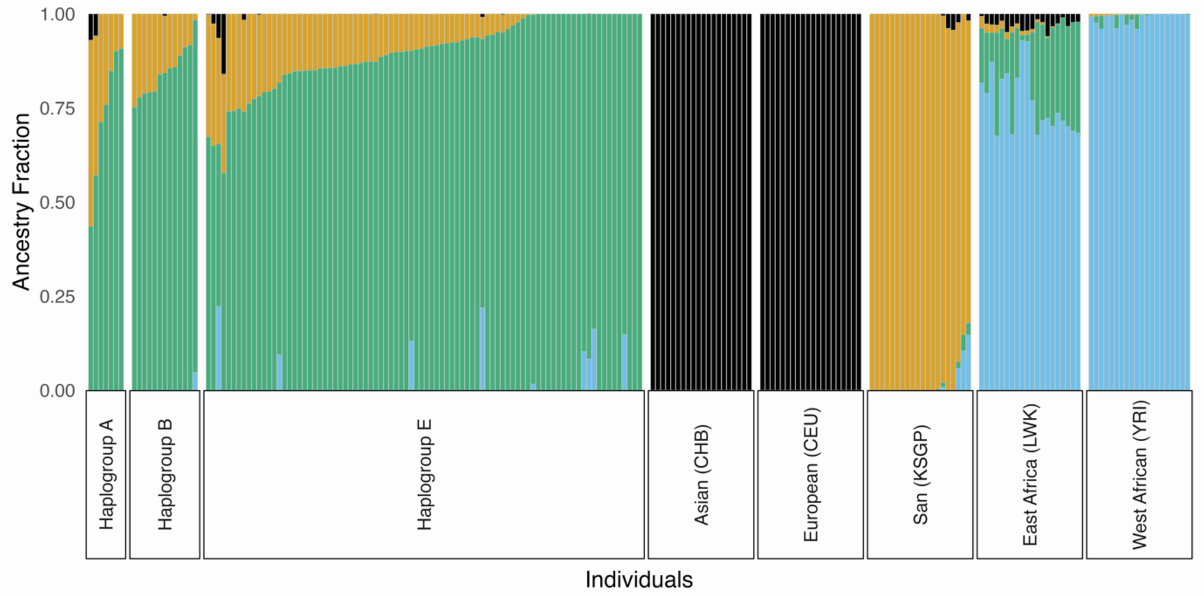

Supplementary Figure 1. Autosomal substructure at K=4 (replicated in 10/10 runs) for African samples predicted to be Y-haplogroups A, B and E, with reference populations CHB, CEU, LWK and YRI from gnomAD v3.1.2[1], and San genomes from the KhoeSan Genome Project[2].

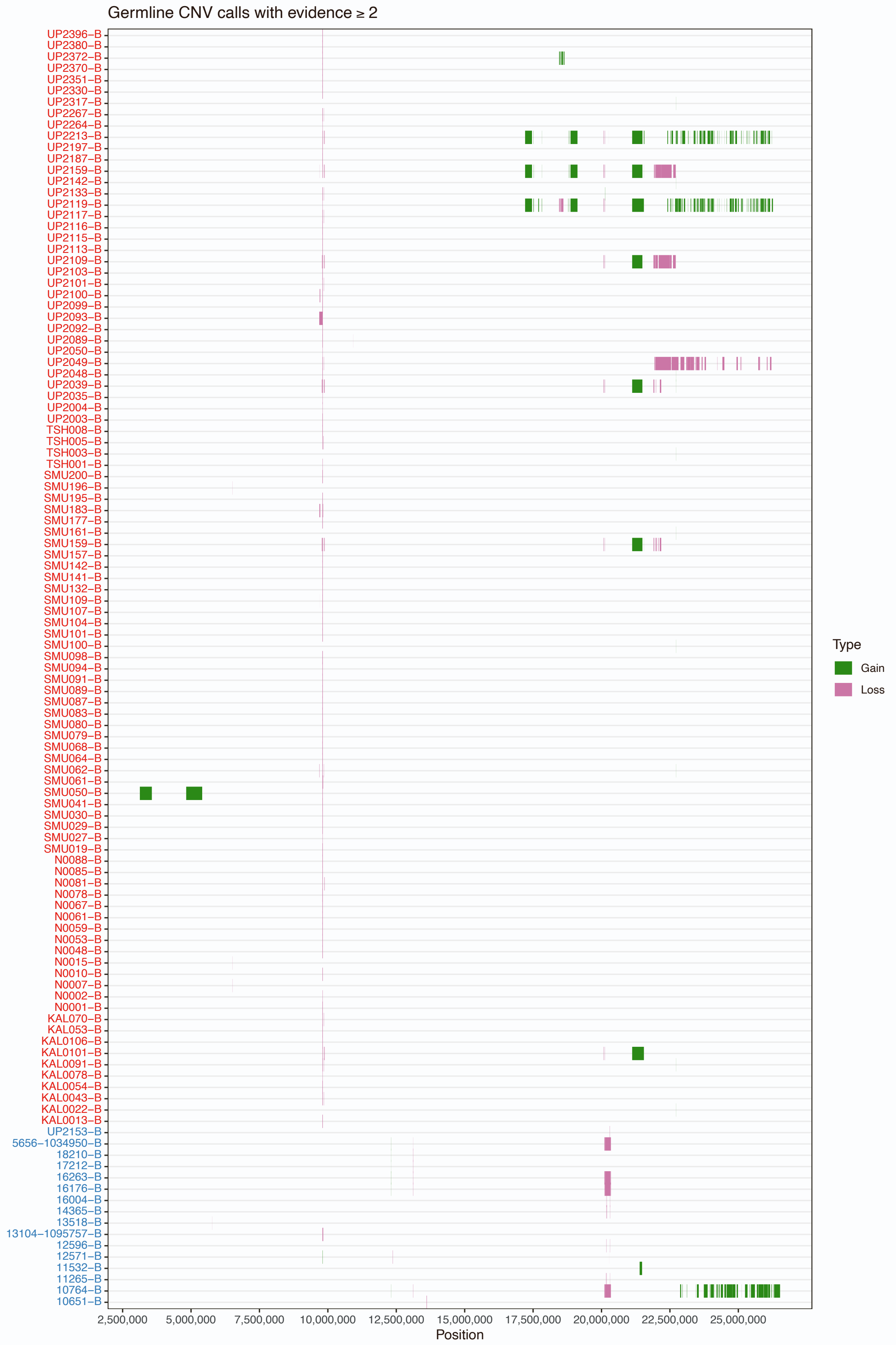

Supplementary Figure 2. Positions of all germline CNV calls with evidence in at least two of three programs (GATK gCNV[3], cn.MOPS[4] and CNVkit[5]). Samples are listed on the y-axis with African samples in red and European samples in blue.

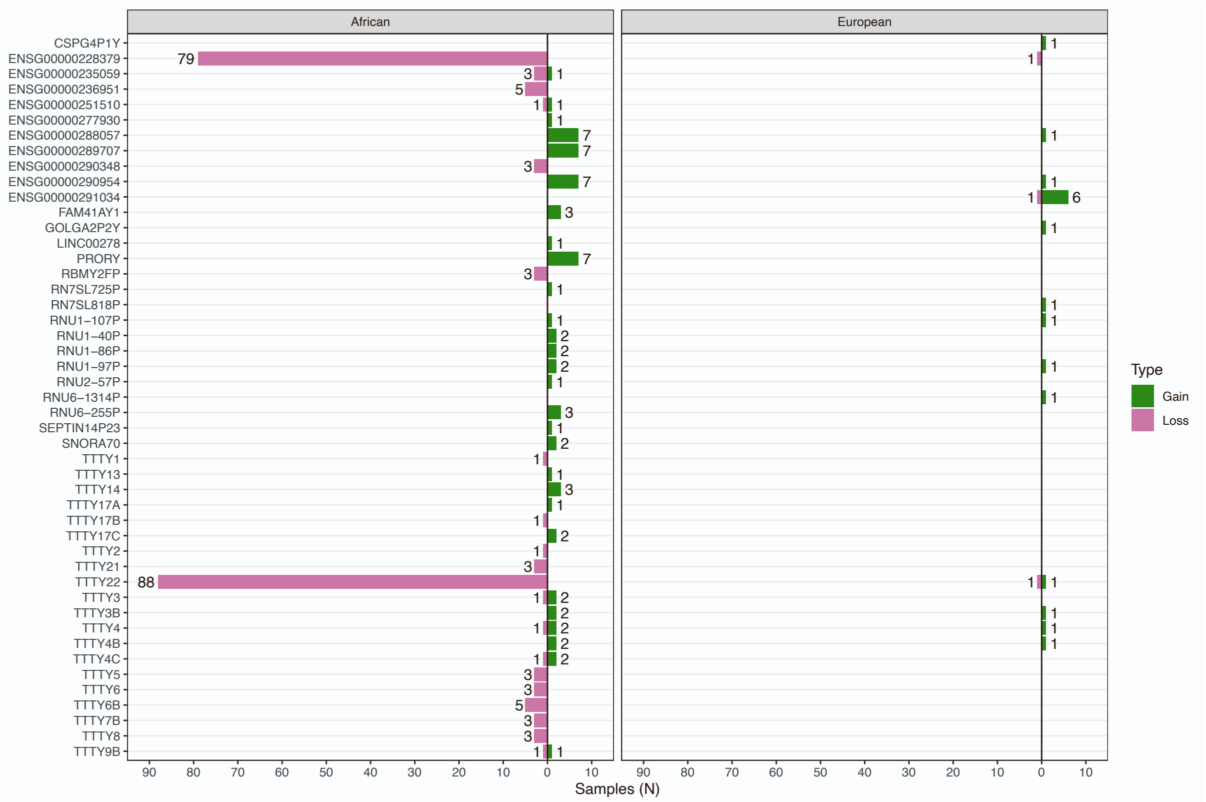

Supplementary Figure 3. The number of samples with a germline copy number gain or loss in RNA genes for each ethnicity.

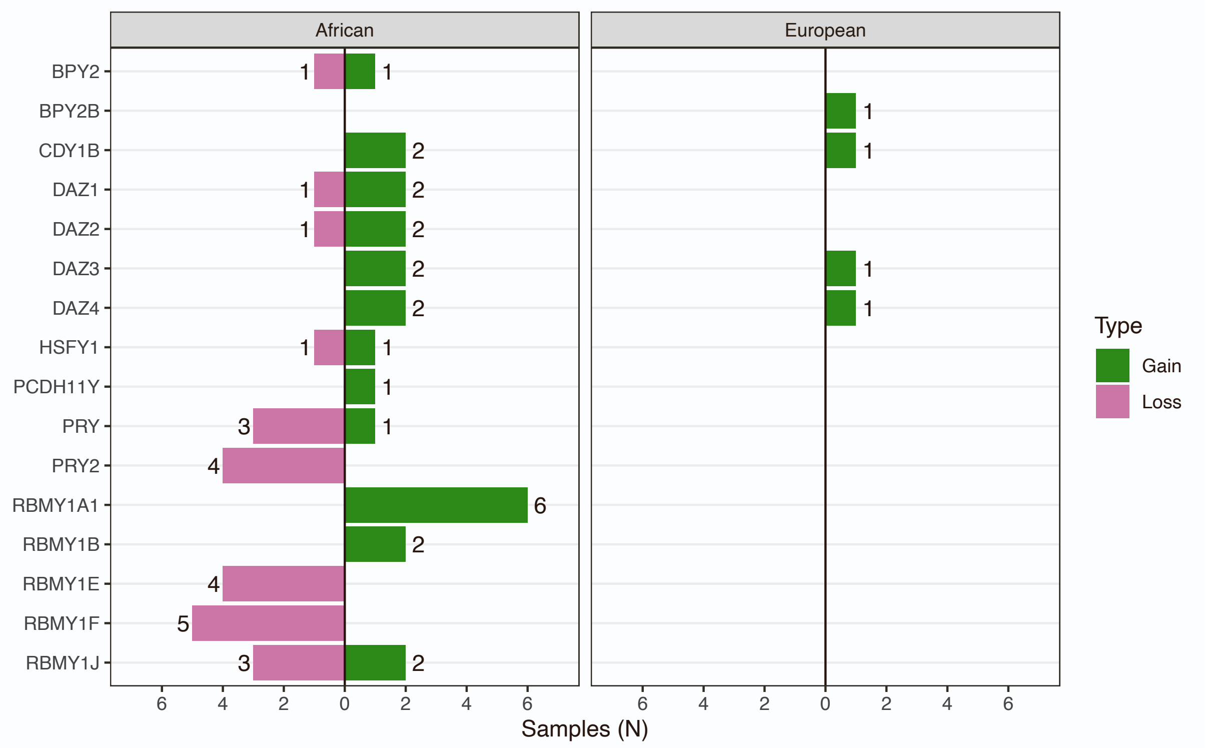

Supplementary Figure 4. The number of samples with a germline copy number gain or loss in protein-coding genes for each ethnicity.

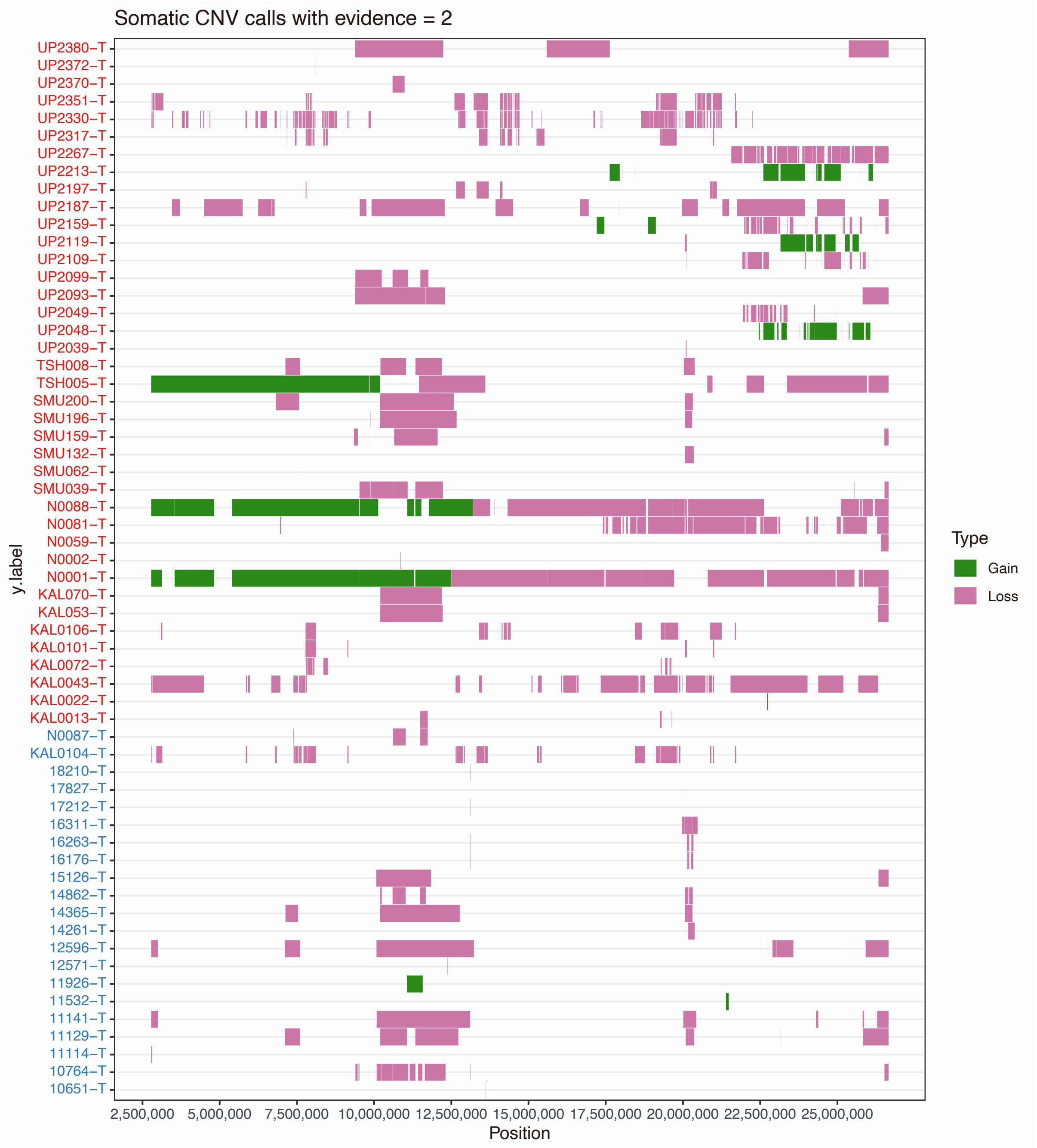

Supplementary Figure 5. Positions of all somatic CNV calls with intersection and consensus between GATK gCNV[3] and CNVkit[5]. Samples are listed on the y-axis with African samples in red and European samples in blue.

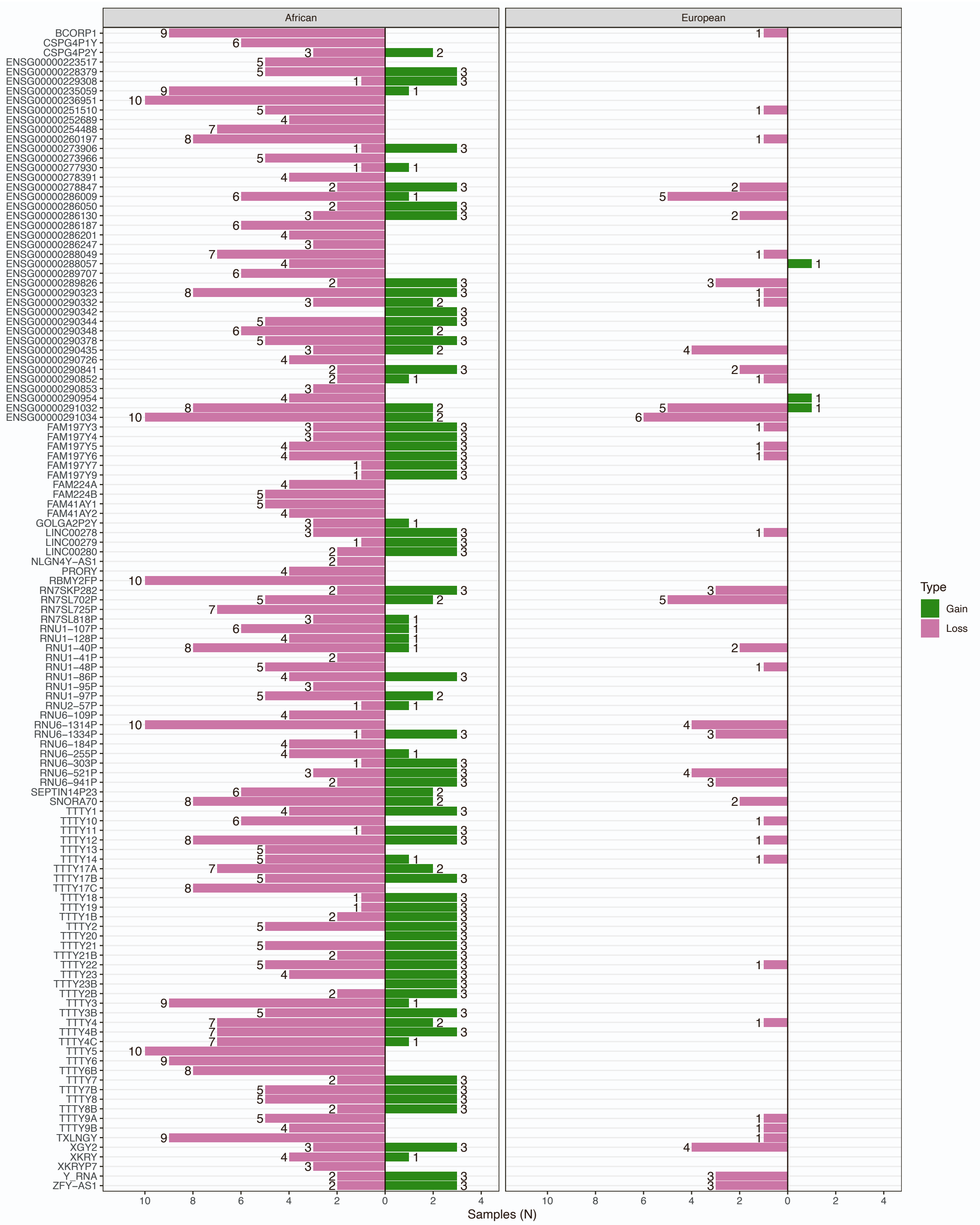

Supplementary Figure 6. The number of samples with a somatic copy number alteration in RNA genes for each ethnicity.

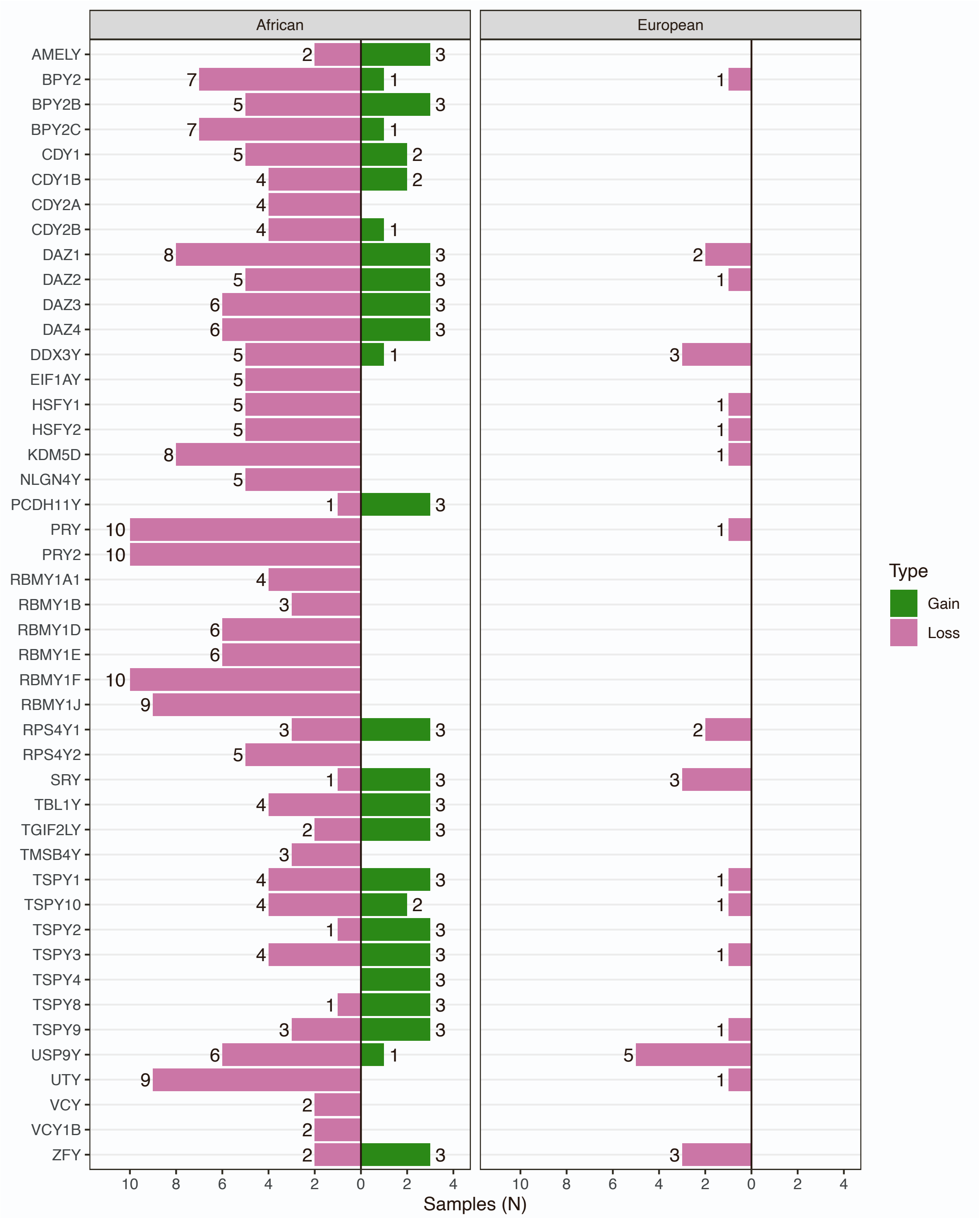

Supplementary Figure 7. The number of samples with a somatic copy number alteration in protein-coding genes for each ethnicity.

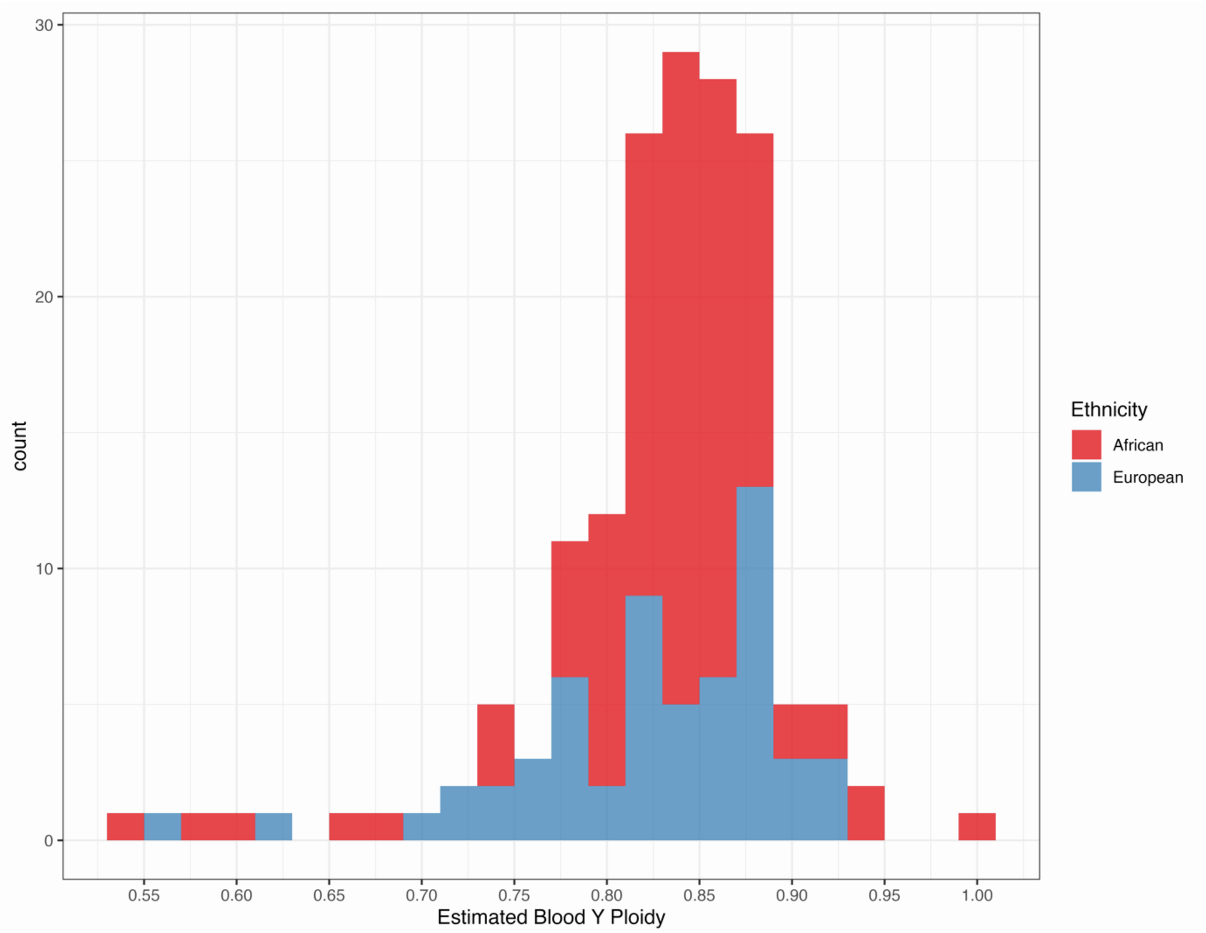

Supplementary Figure 8. Histogram of estimated blood Y ploidy, calculated by dividing mean X-degenerate coverage on chrY by half of the mean whole-genome blood coverage[6].

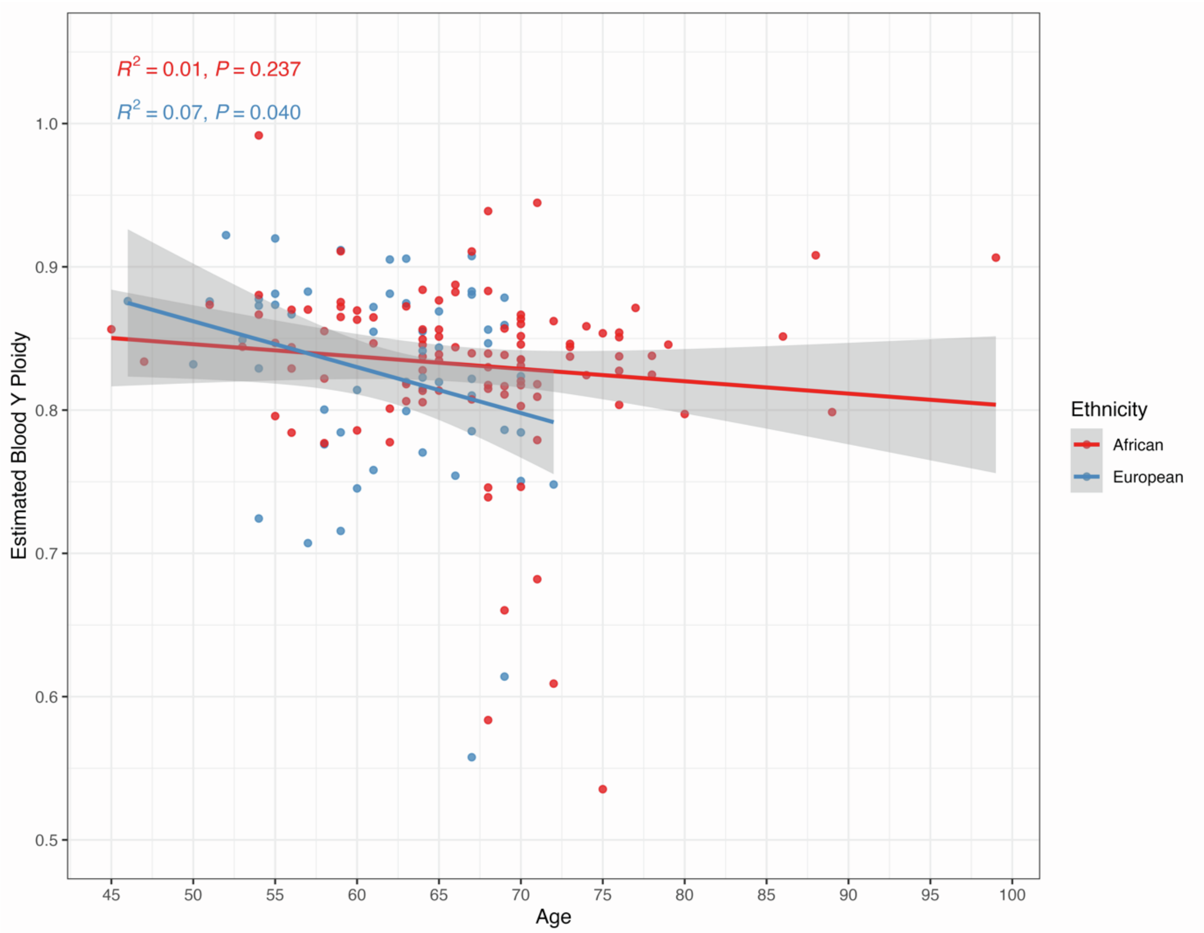

Supplementary Figure 9. Scatter plot of estimated blood Y ploidy against age. P-value and coefficient of determination (R^2) calculated by linear regression. One sample with unknown age was excluded.

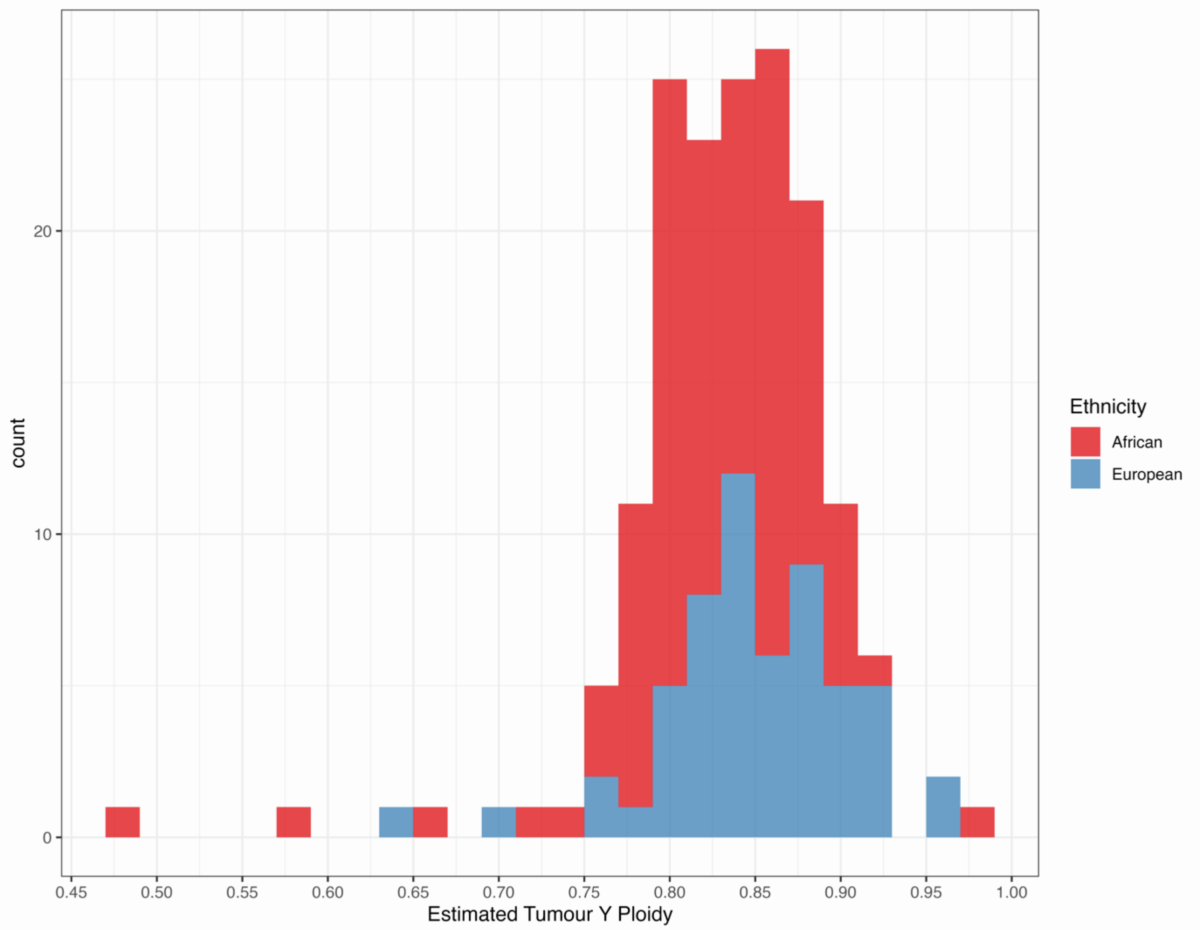

Supplementary Figure 10. Histogram of estimated tumour Y ploidy, calculated by dividing mean X-degenerate coverage on chrY by half of the mean whole-genome tumour coverage[6].

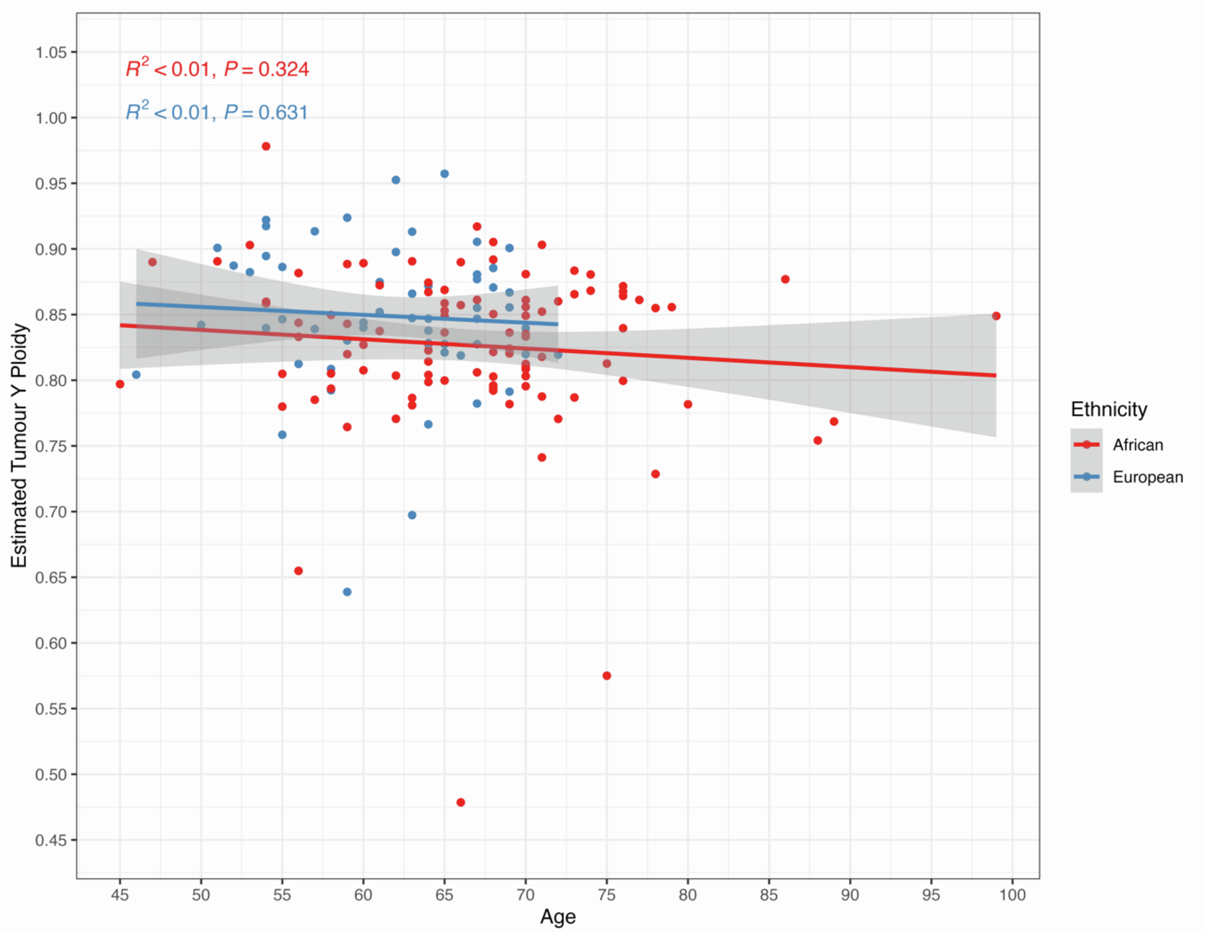

Supplementary Figure 11. Scatter plot of estimated tumour Y ploidy against age. P-value and coefficient of determination (R^2) calculated by linear regression. One sample with unknown age was excluded.

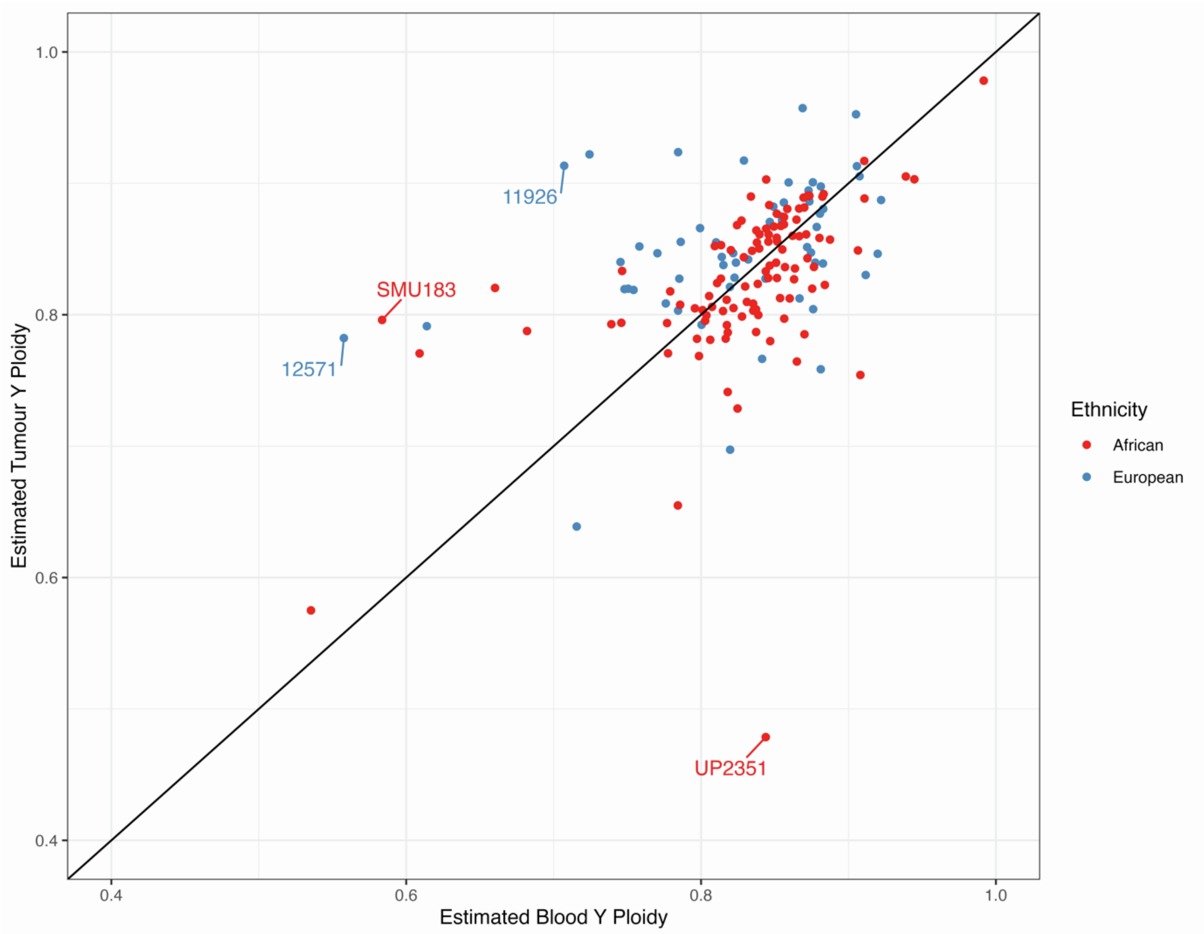

Supplementary Figure 12. Scatter plot of estimated blood *versus* tumour ploidy. Sample UP2351 with a large ploidy loss of 0.36 in the tumour is labelled, along with three samples with >0.2 gain in ploidy.

Supplementary Table 2. Allele frequencies of the alternate allele for germline nonsynonymous variants and ANNOVAR and InterVar predictions. Chr = chromosome, Ref = reference allele, Alt = alternate allele, B = benign, D = damaging/deleterious, P = possibly damaging, T = tolerated, N = neutral, M = medium, L = low, n = number of samples, PDV = potentially deleterious variant.

Position	Ref/ Alt	Gene	avsnp150	REVEL	SIFT pred	SIFT4G pred	Polyphen2 HDIV pred	Polyphen2 HVAR pred	Mutation Taster pred	Mutation Assessor pred
chrY:7064122	G/A	TBL1Y	rs373532788	.	T	T	D	P	D	M
chrY:7074584	A/G	TBL1Y	.	.	T	T	P	B	D	L
chrY:12720687	G/T	USP9Y	rs7067496	.	T	T	B	B	.	L
chrY:12725166	A/G	USP9Y	.	.	T	T	B	B	.	N
chrY:12793155	G/A	USP9Y	.	.	T	T	B	B	.	L
chrY:12842454	C/T	USP9Y	.	.	T	T	P	B	.	L
chrY:12857624	C/G	USP9Y	rs766658730	0.177	D	T	P	B	.	N
chrY:12859400	A/C	USP9Y	.	.	D	T	B	B	.	N
chrY:12914632	G/T	DDX3Y	rs111406208	.	T	T	B	B	.	N
chrY:13305480	G/A	UTY	rs200431840	.	D	D	B	B	P	M
chrY:13355115	T/C	UTY	.	.	D	T	D	D	D	M
chrY:19706182	C/T	KDM5D	rs35681523	.	T	T	B	B	N	N
chrY:19744477	T/A	KDM5D	.	.	D	D	D	D	D	M

Supplementary Table 2 (continued)

Position	Ref/Alt	Gene	FATHMM pred	fathmm-MKL coding pred	PROVEAN pred	MetaRNN pred	CADD phred	DANN score	Intervar	Novel variant	PDV
chrY:7064122	G/A	TBL1Y	T	D	N	T	22.2	0.997	Uncertain significance		PDV
chrY:7074584	A/G	TBL1Y	T	D	D	T	22.1	0.918	Likely pathogenic		PDV
chrY:12720687	G/T	USP9Y	T	D	N	T	33	0.906	Benign		
chrY:12725166	A/G	USP9Y	T	D	N	T	24.4	0.286	Uncertain significance	NOVEL	
chrY:12793155	G/A	USP9Y	T	D	N	T	35	0.527	Uncertain significance		
chrY:12842454	C/T	USP9Y	T	D	N	T	42	0.918	Uncertain significance	NOVEL	PDV
chrY:12857624	C/G	USP9Y	T	D	N	T	32	0.991	Uncertain significance		PDV
chrY:12859400	A/C	USP9Y	T	D	N	T	19.93	0.977	Uncertain significance	NOVEL	PDV
chrY:12914632	G/T	DDX3Y	T	D	N	T	41	0.926	Uncertain significance		
chrY:13305480	G/A	UTY	T	D	N	T	26.1	0.995	Uncertain significance		PDV
chrY:13355115	T/C	UTY	T	D	D	T	23.1	0.996	Uncertain significance	NOVEL	PDV
chrY:19706182	C/T	KDM5D	D	N	N	T	0.472	0.164	Uncertain significance		
chrY:19744477	T/A	KDM5D	T	N	D	T	23.8	0.979	Uncertain significance	NOVEL	PDV

Supplementary Table 2 (continued)

Position	Ref/Alt	Gene	gnomAD v4.1 allele frequency: African/ African American	gnomAD v4.1 allele frequency: European (non-Finnish)	Allele frequency in this study: African (n)	Allele frequency in this study: European (n)	Allele carriers in this study: African patients	Allele carriers in this study: European patients
chrY:7064122	G/A	TBL1Y	0.0021	0.0001	0.066 (7)	0 (0)	N0061-B, SMU030-B, SMU079-B,SMU094-B,TSH008-B,UP2003-B,UP2264-B	
chrY:7074584	A/G	TBL1Y	0.0001	0.0001	0 (0)	0.0175 (1)		11590-B
chrY:12720687	G/T	USP9Y	0.7452	0.0363	1 (106)	0.0702 (4)	All	10651-B, 11114-B,11141-B, 12543-1081384-B
chrY:12725166	A/G	USP9Y	.	.	0.0094 (1)	0 (0)	N0059-B	
chrY:12793155	G/A	USP9Y	0.0001	0.0000	0.0094 (1)	0 (0)	TSH003-B	
chrY:12842454	C/T	USP9Y	.	.	0 (0)	0.0175 (1)		12571-B
chrY:12857624	C/G	USP9Y	0.0000	0.0003	0 (0)	0.0175 (1)		KAL0104-B
chrY:12859400	A/C	USP9Y	.	.	0 (0)	0.0175 (1)		14919-B
chrY:12914632	G/T	DDX3Y	0.0002	0.0000	0.066 (7)	0 (0)	KAL0101-B, SMU159-B, UP2039-B, UP2109-B, UP2119-B, UP2159-B, UP2213-B	
chrY:13305480	G/A	UTY	0.0005	0.0014	0 (0)	0.0175 (1)		10764-B
chrY:13355115	T/C	UTY	.	.	0.0094 (1)	0 (0)	N0081-B	
chrY:19706182	C/T	KDM5D	0.0117	0.0000	0.1226 (13)	0 (0)	KAL0022-B,KAL0072-B,KAL0074-B,N0007-B,N0015-B,N0056-B,N0077-B,N0084-B,SMU039-B,SMU076-B,SMU097-B,SMU196-B,UP2360-B	
chrY:19744477	T/A	KDM5D	.	.	0.0094 (1)	0 (0)	SMU109-B	

Supplementary Table 3. Frequency of each haplogroup in each risk category and Fisher's exact test results, where low risk prostate cancer (LRPCa) is categorised as samples with ISUP grade group of 1 or 2, and high-risk prostate cancer (HRPCa) is categorised as samples with ISUP grade group 3 to 5.

	**LRPCa**, N=25[1]	**HRPCa**, N=138[1]	**p-value**[2]
Major Haplogroup			0.8
A	1 (4.0%)	6 (4.3%)	
B	4 (16%)	9 (6.5%)	
E	13 (52%)	77 (56%)	
G	0 (0%)	1 (0.7%)	
I	2 (8.0%)	8 (5.8%)	
J	1 (4.0%)	5 (3.6%)	
O	0 (0%)	3 (2.2%)	
R	4 (16%)	29 (21%)	
**Europeans (N=57)**			
	**LRPCa**, N=7[1]	**HRPCa**, N=50[1]	**p-value**[2]
Major Haplogroup			>0.9
E	0 (0%)	4 (8.0%)	
G	0 (0%)	1 (2.0%)	
I	2 (29%)	8 (16%)	
J	1 (14%)	5 (10%)	
O	0 (0%)	3 (6.0%)	
R	4 (57%)	29 (58%)	
1 n (%)			
**Africans (N=106)**			
	**LRPCa**, N=18[1]	**HRPCa**, N=88[1]	**p-value**[2]
Major Haplogroup			0.3
A	1 (5.6%)	6 (6.8%)	
B	4 (22%)	9 (10%)	
E	13 (72%)	73 (83%)	

[1] n (%)

[2] Fisher's exact test

Supplementary Table 4. Allele frequencies of somatic nonsynonymous, stop loss, and splice variants and ANNOVAR and InterVar predictions.

Position	Ref/ Alt	Gene	avsnp150	Type	REVEL	SIFT pred	SIFT4G pred	Polyphen2 HDIV pred	Polyphen2 HVAR pred	Mutation Taster pred	Mutation Assessor pred
chrY:6866074	T/A	AMELY	.	Nonstop	.	.	.	.	.	.	.
chrY:6872563	C/T	AMELY	.	Missense	0.064	D	T	D	P	N	L
chrY:7043088	G/A	TBL1Y	.	Missense	.	D	D	D	D	D	M
chrY:7064054	C/T	TBL1Y	rs768766263	Missense	.	T	T	B	B	D	L
chrY:12915228	G/C	DDX3Y	.	Splice site	.	.	.	.	.	.	.
chrY:13479521	T/C	UTY	rs770969453	Missense	.	T	T	B	B	N	N
chrY:20779522	G/A	RPS4Y2	.	Missense	0.337	.	T	B	B	.	H

Supplementary Table 4 (continued)

Position	Ref/Alt	Gene	FATHMM pred	fathmm-MKL coding pred	PROVEAN pred	MetaRNN pred	CADD phred	DANN score	InterVar	Allele frequency in Europeans (n, ID)	Allele frequency in Africans (n, ID)
chrY:6866074	T/A	AMELY	.	D	.	.	22.3	0.749	Uncertain significance	0	0.94% (1, UP2330)
chrY:6872563	C/T	AMELY	.	N	N	D	23.3	0.996	Uncertain significance	0	0.94% (1, SMU080)
chrY:7043088	G/A	TBL1Y	T	D	D	D	23.6	0.994	Likely pathogenic	0	0.94% (1, UP2113)
chrY:7064054	C/T	TBL1Y	T	N	N	T	4.587	0.749	Likely pathogenic	0	0.94% (1, UP2113)
chrY:12915228	G/C	DDX3Y	.	D	.	.	32	0.961	.	0	0.94% (1, UP2330)
chrY:13479521	T/C	UTY	T	N	N	T	6.587	0.427	Uncertain significance	1.75% (1, 11590)	0
chrY:20779522	G/A	RPS4Y2	.	N	.	T	10.16	0.676	Uncertain significance	0	0.94% (1, SMU030)

References

1. Karczewski, K.J., Francioli, L.C., Tiao, G., Cummings, B.B., Alfoldi, J., Wang, Q., Collins, R.L., Laricchia, K.M., Ganna, A., Birnbaum, D.P., et al. (2020). The mutational constraint spectrum quantified from variation in 141,456 humans. Nature *581*, 434-443. 10.1038/s41586-020-2308-7.
2. Jaratlerdsiri, W., Gong, T., Soh, P.X.Y., Jiang, J., Simayi, Z., Petersen, D.C., Holland, E., Chan, E.K.F., Theron, K.E., Haacke, W.H.G., et al. (Under review). A catalogue of early diverged contemporary human genome variation: new insights for ancestrally distinct KhoeSan populations. Nat Comms.
3. Van der Auwera, G., and O'Connor, B.D. (2020). Genomics in the Cloud: Using Docker, GATK, and WDL in Terra, 1st Edition (O'Reilly Media, Inc.).
4. Klambauer, G., Schwarzbauer, K., Mayr, A., Clevert, D.A., Mitterecker, A., Bodenhofer, U., and Hochreiter, S. (2012). cn.MOPS: mixture of Poissons for discovering copy number variations in next-generation sequencing data with a low false discovery rate. Nucleic Acids Res *40*, e69. 10.1093/nar/gks003.
5. Talevich, E., Shain, A.H., Botton, T., and Bastian, B.C. (2016). CNVkit: Genome-Wide Copy Number Detection and Visualization from Targeted DNA Sequencing. PLoS Comput Biol *12*, e1004873. 10.1371/journal.pcbi.1004873.
6. Jaratlerdsiri, W., Jiang, J., Gong, T., Patrick, S.M., Willet, C., Chew, T., Lyons, R.J., Haynes, A.M., Pasqualim, G., Louw, M., et al. (2022). African-specific molecular taxonomy of prostate cancer. Nature *609*, 552-559. 10.1038/s41586-022-05154-6.
